# A dual molecular analogue tuner for dissecting protein function in mammalian cells

**DOI:** 10.1038/ncomms11742

**Published:** 2016-05-27

**Authors:** Ran Brosh, Iryna Hrynyk, Jessalyn Shen, Avinash Waghray, Ning Zheng, Ihor R. Lemischka

**Affiliations:** 1 The Black Family Stem Cell Institute, Icahn School of Medicine at Mount Sinai, New York, New York 10029, USA; 2 Department of Developmental and Regenerative Biology, Icahn School of Medicine at Mount Sinai, 1425 Madison Avenue, 13-20E, New York, New York 10029, USA; 3 Department of Pharmacology and Howard Hughes Medical Institute, University of Washington, Box 357280, Seattle, Washington 98195, USA; 4 The Graduate School of Biomedical Sciences, Icahn School of Medicine at Mount Sinai, New York, New York 10029, USA; 5 Department of Pharmacology and Systems Therapeutics, Icahn School of Medicine at Mount Sinai, New York, New York 10029, USA

## Abstract

Loss-of-function studies are fundamental for dissecting gene function. Yet, methods to rapidly and effectively perturb genes in mammalian cells, and particularly in stem cells, are scarce. Here we present a system for simultaneous conditional regulation of two different proteins in the same mammalian cell. This system harnesses the plant auxin and jasmonate hormone-induced degradation pathways, and is deliverable with only two lentiviral vectors. It combines RNAi-mediated silencing of two endogenous proteins with the expression of two exogenous proteins whose degradation is induced by external ligands in a rapid, reversible, titratable and independent manner. By engineering molecular tuners for NANOG, CHK1, p53 and NOTCH1 in mammalian stem cells, we have validated the applicability of the system and demonstrated its potential to unravel complex biological processes.

## Abstract (web summary)

Loss-of-function approaches are fundamental for dissecting the roles played by genes but methods to simultaneously perturb several proteins in the same mammalian cell are scarce. Here the authors harness the plant auxin and jasmonate hormone-degradation pathways and RNAi technology, to control the levels of two proteins and validate its application in stem cells.

## Graphical abstract

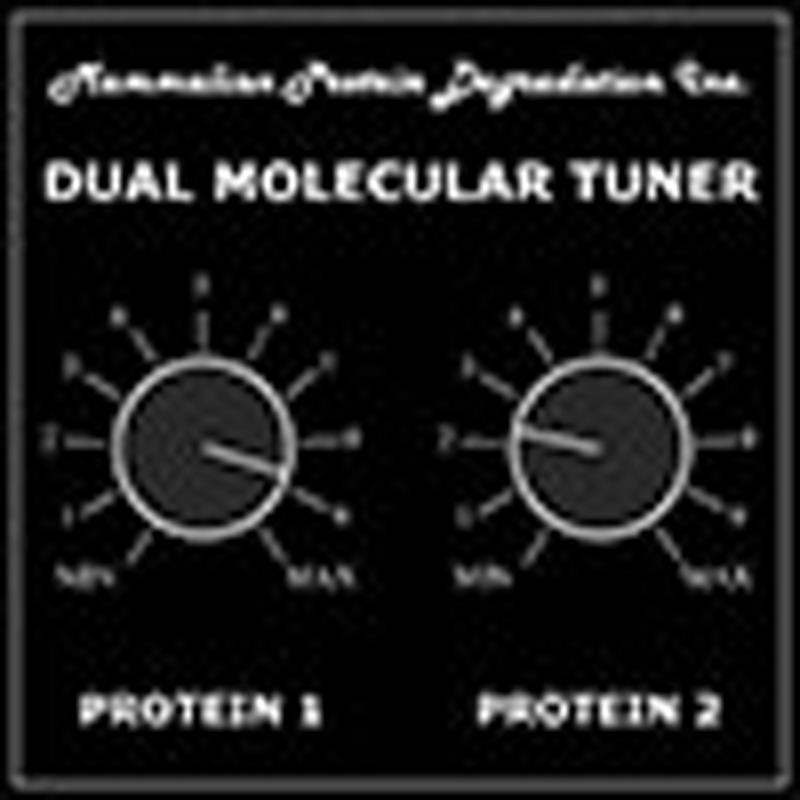

Biologists are increasingly adopting holistic approaches, such as systems biology, to understand life's complexity. Nevertheless, reductionism still remains a primary driving force for scientific progress. Elucidating gene function underlies most biological discoveries and is frequently achieved using loss-of-function analyses. Yet, for mammalian cells in general, and even more so for mammalian stem cells, the biologist's toolbox is limited and primarily includes laborious genomic editing^1^, a limited set of often-nonspecific chemical inhibitors and RNA interference (RNAi). Recently developed tools augment experimental flexibility and accuracy^2^,^3^, but are still limited in applicability, reversibility, titratability, rapidity and multiplicity (Supplementary Table 1). Thus, simple tools for rapid and multiple gene perturbation will facilitate the elucidation of gene functions and molecular networks.

Manipulation of protein levels represents a relatively new loss-of-function approach. To this end, harnessing the plant hormone-induced degradation pathways is particularly attractive due to their efficiency and specificity. The plant hormones auxin (indole-3-acetic acid, IAA) and jasmonate-isoleucine (jasmonic acid-Ile, JA-Ile) bind the intracellular F-Box proteins transport inhibitor response 1 (TIR1) and coronatine insensitive 1 (COI1), respectively, and promote their association with target proteins containing specific degron motifs. TIR1 and COI1, via their F-box domains, assemble into the SCF (SKP1, CUL1 and F-box) E3 ubiquitin–ligase complex, which together with an E2 ubiquitin-conjugating enzyme, catalyses the polyubiquitination and subsequent proteasomal degradation of degron-containing proteins^4^,^5^,^6^,^7^,^8^,^9^. Auxin-bound TIR1 targets proteins containing auxin-induced degradation (AID) degrons, while JA-Ile-bound COI1 targets proteins containing JAZ degrons (Fig. 1a). Nishimura *et al*.^10^ developed a system enabling conditional protein regulation by adapting the auxin-induced degradation pathway to non-plant cells. They reported that ectopic TIR1 can mediate auxin-dependent degradation of AID-fused proteins and demonstrated the system's feasibility with a simple plasmid (pAID) harbouring a cytomegalovirus promoter-driven polycistronic mRNA encoding TIR1 and a plant protein carrying the AID degron. Fusing a protein-of-interest (POI) to the degron enabled the degradation of the POI following auxin treatment^10^. Despite its simplicity, pAID has major limitations in terms of applicability to mammalian cells. These include a viral promoter prone to silencing in embryonic stem cells (ESCs)^11^,^12^, a lack of a designated selectable marker, the inability to suppress endogenous genes and a large degron (228 AAs) liable to interfere with the POI's function. For these and other reasons (Supplementary Table 1), this technology has been primarily applied to yeast, where endogenous genes are easily disrupted and pAID-carrying clones are readily isolated. Of note, although auxin-dependent degradation was previously used to study mammalian cells, its implementation required multiple consecutive genetic manipulations and was mainly confined to cancer cell lines^13^,^14^,^15^,^16^. In recent times, auxin-dependent degradation was also harnessed *in vivo* to study *Caenorhabditis elegans*^17^.

Mammalian ESCs have gained much interest as a model for developmental biology and a therapeutic avenue. ESCs are unique in their unlimited self-renewal and pluripotency, a state maintained by a transcription factor network revolving around SOX2, OCT4 (POU5F1) and NANOG^18^. Combining loss-of-function and genetic complementation (rescue) strategies, we broadened and characterized the ESC self-renewal network^18^,^19^,^20^,^21^,^22^. Nevertheless, we sought to develop an improved experimental system that upgrades the stem cell biologist's toolbox and facilitates faster, tighter and combinatorial dissection of gene and protein function.

Here we report a mammalian dual-protein rescue system that harnesses the auxin and JA-Ile pathways, and is specifically tailored to ESCs. For each hormone, we engineered a lentiviral vector harbouring a short hairpin RNA (shRNA), a hormone receptor, a short degron and a selectable marker. Using a two-step cloning protocol, each vector is easily modified to contain the desired shRNA and degron-fused POI, which enables silencing of a gene-of-interest and its replacement by a POI whose degradation is induced by the appropriate hormone. The combination of these two vectors offers simultaneous control over two proteins in the same cell. By applying this system to study key ESC decision-making proteins, such as NANOG, CHK1, p53 and NOTCH1, we have demonstrated the system's potential to facilitate experimental designs that were previously unfeasible or overcomplicated.

## Results

### pRAIDRS functions as an auxin-induced degradation rescue system

**Figure 1:**
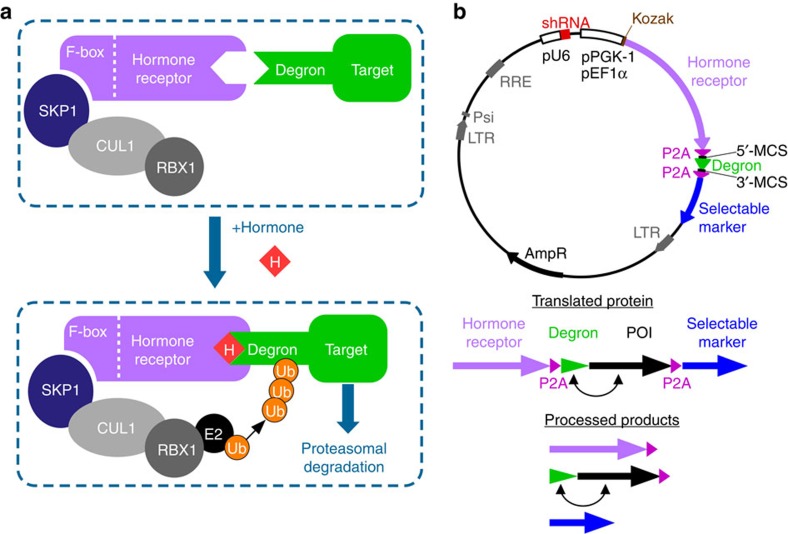
Mechanism of plant hormone-induced degradation and structure of vectors. (**a**) Schematic illustration of plant hormone-induced protein degradation pathways. The plant SCF E3 ubiquitin ligase complex comprises SKP1, CUL1 and an F-box hormone receptor. On binding its cognate hormone, the receptor recruits the SCF complex to a target protein containing a degron motif. A recruited E2 ubiquitin-conjugating enzyme ubiquitinates the target, leading to its rapid proteasomal degradation. H, hormone; Ub, ubiquitin. (**b**) Upper part, schematic representation of the pRAIDRS and pJAZ vector structure. Bottom part, pre- and post-P2A-mediated processing of the translated components. The two-headed arrow indicates that the degron can be fused to either terminus of the POI. AmpR, ampicillin resistance β-lactamase; LTR, long-terminal repeat; MCS, multiple cloning site; POI, protein-of-interest; Psi, Psi packaging signal; RRE, Rev response element. See also Supplementary Fig. 1.

We aimed at designing a vector that enables depletion of an endogenous gene-of-interest and its replacement by an exogenous POI whose degradation is induced by auxin. This approach represents a genetic complementation (rescue) system, in which a phenotype exerted by silencing a gene-of-interest is conditionally reversed by exogenous expression of that gene product. To this end, we engineered pRAIDRS (RNAi and auxin-induced degradation rescue system), a lentiviral vector containing all elements for construction of an auxin-regulated rescue system. As depicted in Fig. 1b, a U6 promoter drives the expression of an shRNA that silences an endogenous gene-of-interest. A second promoter, either phosphoglycerate kinase-1 (pPGK-1) or the stronger elongation factor 1α (pEF1α) (Supplementary Fig. 1a), followed by a Kozak sequence, drives the expression of an mRNA encoding three in-frame proteins separated by two porcine teschovirus-1 2A (P2A) peptides. The first protein is a codon-optimized *Oryza sativa* (rice) TIR1 auxin receptor (*Os*TIR1). The second component is a shortened AID degron derived from *Arabidopsis thaliana* IAA17 (*At*IAA17), which can be fused to either terminus of the POI. The last component is a selectable marker, either puromycin N-acetyl-transferase (PuroR) or blasticidin-*S* deaminase (BSD), conferring puromycin or blasticidin resistance, respectively. Mammalian cells transduced with pRAIDRS express *Os*TIR1, which associates with SKP1 and forms a functional SCF^TIR1^ complex^10^. Following auxin treatment, SCF^TIR1^ mediates degron polyubiquitination, leading to degradation of the POI (Fig. 1a).

The full-length *At*IAA17, originally used in pAID (ref 10), is imperfect as a degron due to its large size (228 AAs), its propensity to confer nuclear localization^23^ and other potentially undesirable activities it possesses as a plant transcription factor. Therefore, we mapped the minimal required AID degron to a 47-AA region (AID^47^) spanning *At*IAA17 residues 63–109 (Supplementary Fig. 1b,c), which mostly overlaps with a previously reported shortened AID degron^24^. Notably, we observed that in pRAIDRS-transfected HEK-293T cells, green fluorescent protein (GFP) is spontaneously cleaved from the full-length *At*IAA17 degron (AID^228^), but not from AID^47^ (Supplementary Fig. 1c,d), suggesting that a shorter degron might also be more cleavage resistant. However, as other labs who have used AID^228^ did not report spontaneous cleavage, this phenomenon might be specific to our cell lines, POI or vector architecture. We next compared the degradation of cytoplasmic and nuclear POIs by analysing the effect of a nuclear localization signal (NLS) on the degradation of GFP-AID^47^, and found both highly effective, but NLS-GFP-AID^47^ degradation faster (Supplementary Fig. 1e,f).

### pRAIDRS enables rapid and titratable conditional regulation

**Figure 2:**
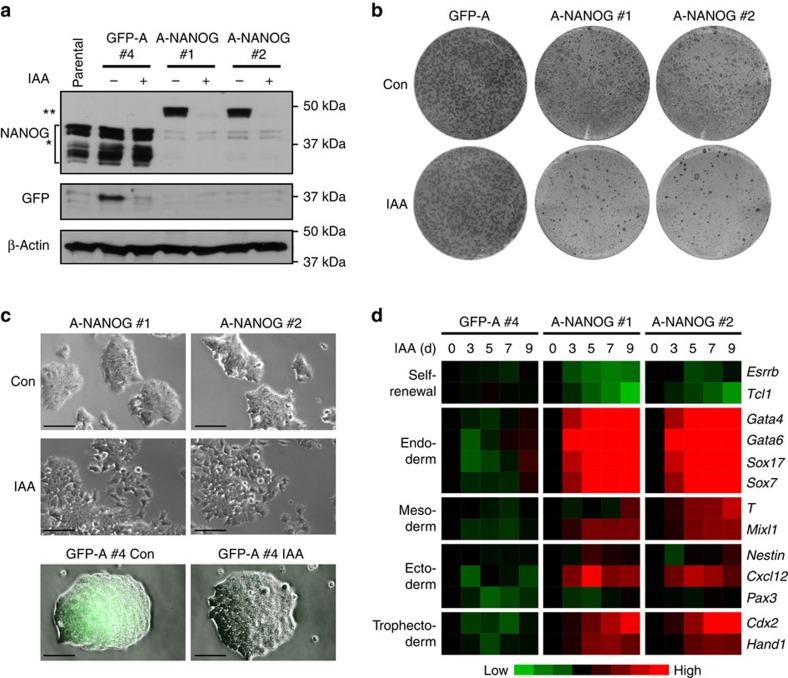
An auxin-degradable NANOG rescue system in mESCs. CCE mESCs were infected with pRAIDRS AID^47^-NANOG (A-NANOG) or GFP-AID^47^ (GFP-A) and selected clones with ESC morphology were analysed for the effect of auxin (IAA) treatment. (**a**) A western blot analysis depicting endogenous NANOG (*) and A-NANOG (**) in parental mESCs, and in the indicated clones. β-Actin serves as a loading control. Experiment was repeated three times and a representative blot is presented. (**b**) mESC clones were plated at low density, grown in the presence of ethanol (Con) or auxin (IAA) for 3–4 days and assayed for AP activity. GFP-A mESCs reached the desired confluency a day earlier and therefore the images were taken on different days. (**c**) Upper part: bright-field images showing representative morphology of A-NANOG mESCs following 3 days of ethanol (Con) or auxin (IAA) treatment. Lower part: merged bright-field and GFP fluorescence images of ethanol or auxin-treated GFP-A mESCs. Scale bars, 100 μm. (**d**) mESC clones were treated with auxin for the indicated number of days. All cells were subjected to the same concentration of ethanol for the duration of experiment. Quantitative real-time PCR analysis was performed for selected self-renewal and differentiation markers, and normalized expression levels are represented as a heatmap. (**b**–**d**) Differentiation experiment was repeated two times and representative results are displayed. See also Supplementary Figs 2 and 9.

To demonstrate the applicability of pRAIDRS as a rescue system in mammalian stem cells, we engineered mouse ESCs (mESCs) in which the protein level of NANOG is controlled by auxin. We infected mESCs with pRAIDRS harbouring an shRNA targeting the 3′-untranslated region (3′-UTR) of *Nanog* mRNA and an AID^47^-fused *Nanog* coding sequence (A-NANOG) lacking UTRs. As a control, mESCs were infected with pRAIDRS containing only GFP-AID^47^ (GFP-A). Post-selection clones demonstrated effective silencing of endogenous NANOG by the shRNA, whereas exogenous A-NANOG, which was expressed at levels comparable to endogenous NANOG in control cells, was effectively and rapidly depleted following auxin treatment (Fig. 2a and Supplementary Fig. 2a). Phenotypically, auxin treatment of A-NANOG mESCs, but not GFP-A mESCs, resulted in depletion of alkaline phosphatase (AP) positive colonies, loss of ESC morphology and a transcriptional programme characteristic of NANOG inactivation^22^, namely downregulation of self-renewal genes and induction of endodermal differentiation markers (Fig. 2b–d and Supplementary Fig. 2b). A similar transcriptional response was elicited by shRNA-mediated NANOG depletion (Supplementary Fig. 2c,d). In contrast, mESCs infected with pRAIDRS harbouring a *Nanog* shRNA and a *Nanog* coding sequence fused to an irrelevant degron (*Os*JAZ^33^, see below) did not respond to auxin treatment (Supplementary Fig. 2e,f). These results demonstrate the applicability of pRAIDRS as a molecular switch that facilitates dissection of protein function in mESCs.

**Figure 3:**
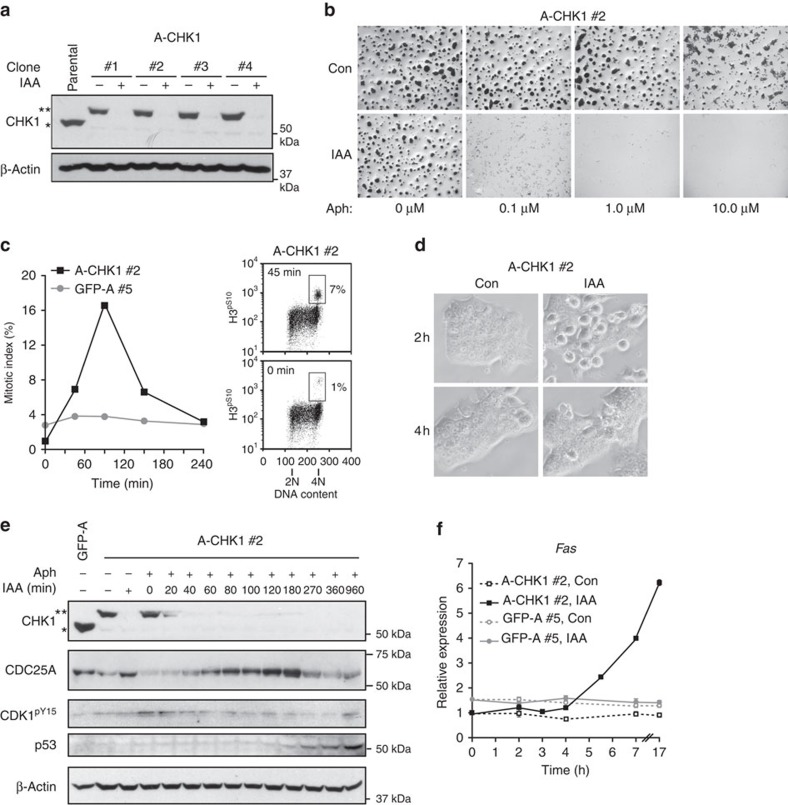
Rapid CHK1 depletion sensitizes mESCs to DNA damage. (**a**) A western blot analysis depicting endogenous CHK1 (*) and AID^47^-CHK1 (A-CHK1, **) in four selected clones and in parental non-infected mESCs. β-Actin serves as a loading control. Experiment was repeated three times for clones #1 and #2, and a representative blot is presented. (**b**) A-CHK1 #2 mESCs were treated with ethanol (Con) or auxin (IAA) for 1 day. Cells were then treated with the indicated concentrations of aphidicolin (Aph). Equal concentrations of DMSO were applied to all conditions. The next day, cells were stained with crystal violet and plates were scanned. Experiment was repeated three times and a representative result is displayed. (**c**) mESC clones were pretreated with 1 μM aphidicolin for 1 day and were then treated with auxin for the indicated time periods. Left panel: mitotic index was calculated as the percentage of H3^pS10^-positive cells with 4N DNA content, measured by flow cytometry. Right panel: dot plots for A-CHK1 #2 mESCs treated with auxin for 0 or 45 min. Mitotic cells are gated. (**d**) Cells were treated as in **c**. Bright-field microscope images showing synchronous cell rounding, a feature of late mitotic cells, 2 h following auxin treatment in aphidicolin-treated A-CHK1 #2 cells. (**e**) Cells were treated as in **c** and subjected to a western blot analysis. Tyr15 phosphorylation of CDK1 (CDK1^pY15^) was detected using a phospho-specific antibody. β-Actin serves as a loading control. (**f**) Quantitative real-time PCR analysis of *Fas* mRNA in cells treated as described in **c**. Error bars represent s.d. of three technical replicates. (**c**–**f**) Kinetic experiment was repeated three times and representative results are displayed. See also Supplementary Figs 3,4,5 and 9.

To exemplify the rapidity of degradation enabled by pRAIDRS, we established a rescue system for the checkpoint kinase CHK1 in mESCs. CHK1 is required for mouse development and its disruption severely impairs DNA damage responses^25^,^26^. Multiple roles are also attributed to CHK1 in normal cell cycle progression^27^,^28^ and in mESC self-renewal^20^. We infected mESCs with pRAIDRS harbouring a *Chk1* 3′-UTR-targeting shRNA and an AID^47^-fused *Chk1* coding sequence (A-CHK1). A western blot analysis of selected clones demonstrated efficient silencing of endogenous CHK1 and complete auxin-dependent degradation of A-CHK1 (Fig. 3a). Next, A-CHK1 cells were monitored for the effects of CHK1 depletion. When cells were infected and selected in the presence or absence of auxin, a marked auxin-dependent depletion of AP-positive colonies was observed (Supplementary Fig. 3a), apparently supporting the reported roles of CHK1 in mESC self-renewal. However, CHK1 depletion in post-selection cells had only a marginal effect, if any, on proliferation rate, stage specific embryonic antigen-1 (SSEA-1) levels, mRNA expression patterns or apoptosis (Supplementary Figs 3 and 4). These data imply that the initial effect of CHK1 depletion may reflect its role during cellular stress responses induced by viral infection or drug selection.

We then used pRAIDRS to study the role of CHK1 in the mESC DNA damage response. To this end, cells were treated with aphidicolin, a DNA polymerase inhibitor that induces DNA breaks and activates the ATR-CHK1 pathway^29^. CHK1 depletion dramatically sensitized mESCs to aphidicolin, as auxin-treated A-CHK1 cells died following treatment with 0.1 μM aphidicolin, whereas control cells survived following treatment with 100-fold higher concentrations of aphidicolin (Fig. 3b). This hypersensitivity was specific to CHK1 depletion as auxin- and control-treated GFP-A cells responded indistinguishably to aphidicolin treatment (Supplementary Fig. 4a,b). CHK1 depletion in aphidicolin-treated cells resulted in rapid induction of apoptosis, activation of a p53 (TRP53) transcriptional response, predominantly of the p53 target *Fas* that encodes a death receptor^30^, as well as a later induction of differentiation (Supplementary Fig. 4c–f). We hypothesized that the aphidicolin susceptibility of CHK1-depleted cells stems from the ability of CHK1 to phosphorylate and induce the cytoplasmic sequestration or degradation of CDC25 phosphatases, which, in turn, augments the inhibitory Tyr15 phosphorylation of CDK1 (CDK1^pY15^), preventing cell cycle progression^31^. Indeed, rapid (20 min) auxin-dependent depletion of CHK1 in aphidicolin-treated mESCs resulted in synchronous mitotic entry 45–90 min post-auxin treatment, parallelling CDC25A stabilization and the decrease in CDK1^pY15^, and preceding p53 stabilization and the induction of *Fas* mRNA (Fig. 3c–f and Supplementary Fig. 4g). Thus, depleting CHK1 in DNA-damaged mESCs led to a series of consecutive phenotypes already observable 45 min post treatment. Moreover, by titrating down CHK1 levels in DNA-damaged mESCs, we demonstrated pRAIDRS applicability as a sensitive analogue tuner that enables fine-tuning of protein levels and their associated phenotypes (Supplementary Fig. 5), facilitating in-depth analyses of protein dose responses.

**Figure 4:**
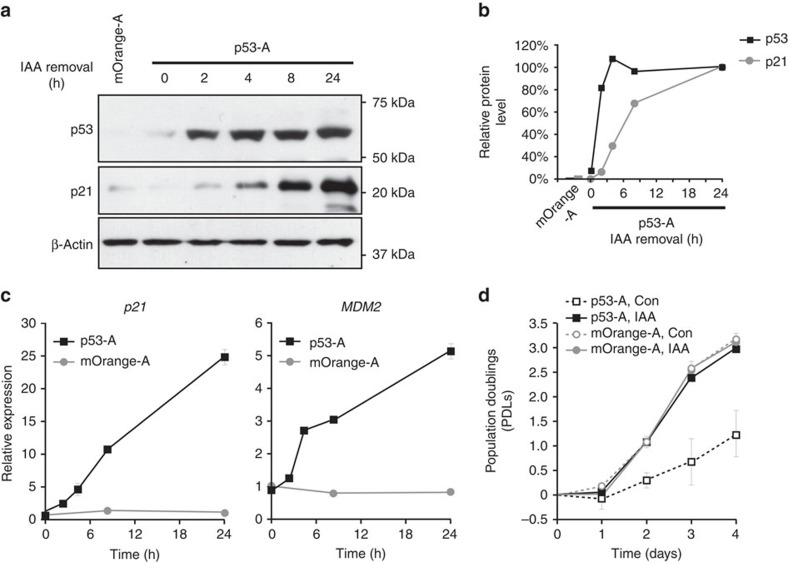
pRAIDRS enables reversible regulation of protein level. NCI-H1299 p53-null lung adenocarcinoma cells were infected with pRAIDRS p53-AID^47^ (p53-A) or mOrange-AID^47^ (mOrange-A) as a control. Cells were maintained with 200 μM auxin, to constantly induce p53 degradation. (**a**) Cells were washed three times, incubated with fresh media in the absence of auxin for the indicated time periods and subjected to a western blot analysis of p53 and p21. β-Actin serves as a loading control. (**b**) Quantification of protein levels (presented in **a**). Values were normalized such that the level of each protein at the 24-h time point was set to 100%. (**c**) Cells were treated as described above and subjected to a quantitative real-time PCR analysis of the p53 target genes p21 (*CDKN1A*) and *MDM2*. Error bars represent s.d. of three technical replicates. Experiment was repeated three times and representative results are displayed. (**d**) Cells were grown in the presence of ethanol (Con) or auxin (IAA) and counted every day for 4 days. Media was replaced daily. Population doublings (PDLs) were calculated as Log_2_(cell output/cell input). Error bars represent s.d. of three technical replicates. Experiment was repeated twice and representative results are displayed. See also Supplementary Fig. 9.

Auxin-induced degradation was shown to be reversible^10^. To demonstrate this for pRAIDRS, we engineered p53-null lung adenocarcinoma cells (NCI-H1299) expressing an auxin-degradable wild-type p53-AID^47^ (p53-A). These cells were infected and cultured in the presence of auxin to prevent the stabilization of p53, known for its ability to inhibit cell growth^32^,^33^. However, following auxin removal p53 was rapidly stabilized, leading to the induction of the p53 target genes *p21* (*CDKN1A*) and *MDM2*, and resulting in growth retardation (Fig. 4). In sum, these data validate and exemplify pRAIDRS as an easy-to-use single-vector system enabling the construction of highly rapid, titratable, reversible and non-stressful molecular tuners in mESCs and other cell types.

### pJAZ functions as a coronatine-induced degradation rescue system

Simultaneous conditional regulation of two proteins represents a powerful tool for complex analyses. We therefore sought to engineer a second rescue system that harnesses the plant jasmonate-induced degradation response. As described above, in plants, isoleucine-conjugated jasmonate (JA-Ile) mediates the binding of the F-box hormone receptor COI1 and the JAZ degron domain of target proteins, which are consequently ubiquitinated and degraded^9^,^30^ (Fig. 1a). We speculated that expression of COI1 in mammalian cells would enable hormone-dependent degradation of JAZ-fused POIs. As mammalian cells lack the pathway for JA-Ile conjugation, we used coronatine, a bacterial analogue of JA-Ile^34^. Using the same architecture as pRAIDRS (Fig. 1b), we constructed pJAZ, a vector harbouring a codon-optimized *A. thaliana* COI1 receptor (*At*COI1) and a 23-AA JAZ degron (*At*JAZ^23^, Supplementary Fig. 6a) that we have previously identified as the *A. thaliana* JAZ1 minimal degron motif^5^.

**Figure 5:**
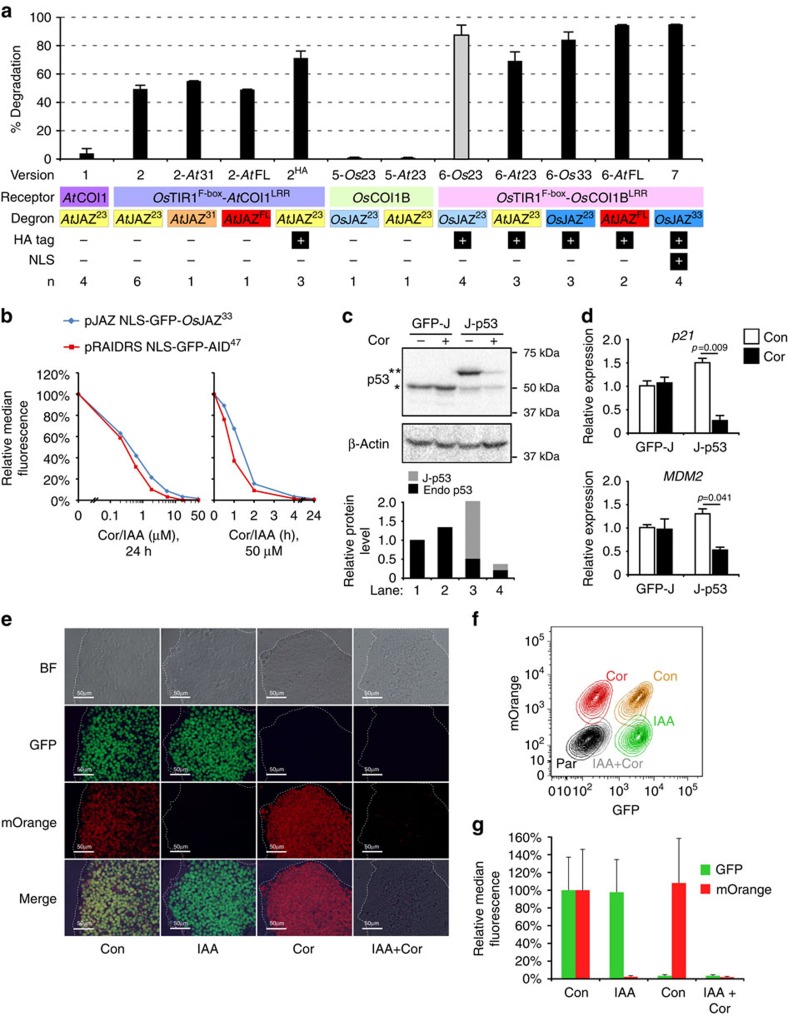
A coronatine-induced degradation rescue system. (**a**) HEK-293T cells were infected with the indicated pJAZ versions, selected and treated for 1 day with coronatine. GFP fluorescence was measured by flow cytometry and % degradation was calculated as described in Methods. The corresponding components and biological replicate number (*n*) are indicated. The bar for pJAZ 6-*Os*23 is in grey colour, to indicate reduced GFP fluorescence in non-treated cells (Supplementary Fig. 6f). (**b**) HEK-293T cells were infected with pRAIDRS NLS-GFP-AID^47^ or pJAZ (version 7) NLS-GFP-*Os*JAZ^*33*^, treated with the indicated concentrations of the corresponding hormone for 24 h (left panel) or with 50 μM of hormone for the indicated time periods (right panel). GFP fluorescence was measured using flow cytometry. Experiment was repeated three times and representative results are presented. (**c**,**d**) H9 hESCs were infected with pJAZ NLS-GFP-*Os*JAZ^33^ (GFP-J) or pJAZ *Os*JAZ^33^-p53 (J-p53) that harbours an shRNA targeting the 3′-UTR of p53 and an *Os*JAZ^33^ degron-fused p53 coding sequence lacking UTRs. Selected hESCs were treated with 50 μM coronatine (Cor) or 0.1% DMSO (Con) for 1 day. A western blot analysis (**c**, upper panel) and protein level quantification (**c**, lower level) demonstrate knockdown of endogenous p53 (*) and expression of J-p53 (**), as well as effective (90%) coronatine-dependent degradation of J-p53. Quantitative real-time PCR analysis (**d**) for p53 target genes. Error bars represent s.d. of three technical replicates. *P*-values were calculated using unpaired Student's *t*-test. Experiment was repeated twice and representative results are displayed. (**e**–**g**) H9 hESCs were infected with pJAZ NLS-GFP-*Os*JAZ^33^ (harbouring PuroR) and pRAIDRS NLS-mOrange-AID^47^ (harbouring BSD), selected and cloned. Cells were treated with either ethanol and DMSO (Con), auxin and DMSO (IAA), ethanol and coronatine (Cor) or auxin and coronatine (IAA+Cor). After 24 h, microscopic bright-field (BF) and fluorescence images were taken (**e**, scale bars, 100 μm) and cells were subjected to flow analysis (**f**, contour plots; **g**, quantification, error bars represent s.d.). Parental cells (Par) are presented as autofluorescence control. Experiment was repeated three times and representative results are displayed. See also Supplementary Figs 6,7 and 9.

For initial testing, we infected HEK-293T cells with pJAZ harbouring GFP-*At*JAZ^23^ and treated them with coronatine. Disappointingly, GFP degradation was extremely ineffective (Fig. 5a, version 1). We then systematically and iteratively optimized pJAZ by testing different COI1 orthologues and fusion proteins, and by altering the degron length and origin (Fig. 5a and Supplementary Fig. 6). We hypothesized that the lack of coronatine-dependent degradation stems from insufficient binding of *At*COI1 to human SKP1 (*Hs*SKP1). We therefore generated an *Os*TIR1^F-box^-*At*COI1^LRR^ chimera composed of *Os*TIR1 F-box domain (AA 1–39)^4^, which binds *Hs*SKP1 effectively^10^, and *At*COI1 leucine-rich repeat (AA 52–592), the receptor region responsible for hormone and degron binding^5^. Cells infected with pJAZ version 2 demonstrated ∼50% coronatine-dependent GFP degradation. A similar chimeric receptor harbouring *Hs*SKP2^F-box^ (version 3) and various *At*COI1-*Hs*SKP1 fusions (versions 4a-d) failed to mediate coronatine-dependent degradation. We next tested an extended 31-AA degron (*At*JAZ^31^), as well as *At*JAZ^FL^, the full-length *A. thaliana* JAZ1 protein, and found that neither enhanced pJAZ function. To test whether the *Os*TIR1^F-box^-*At*COI1^LRR^ receptor is sufficiently expressed, we added an amino-terminal haemagglutinin (HA) tag and found the receptor level comparable to the level of HA-*Os*TIR1 in pRAIDRS-infected cells (Supplementary Fig. 6e) and, hence, presumably sufficient. Unexpectedly, the HA tag boosted pJAZ efficiency to ∼70% (version 2^HA^), possibly by stabilizing the receptor^24^. We next reasoned that at 37 °C, a rice coronatine receptor (*Os*COI1) might function better than *At*COI1, as reported for the auxin receptor^10^. Of the three *Os*COI1 paralogues, we chose *Os*COI1B, as it binds a larger variety of JAZ proteins^35^, and tested it with either the *At*JAZ^23^ degron or with a 23-AA rice degron, *Os*JAZ^23^ (Supplementary Fig. 6a). We found both versions (5-*At*23 and 5-*Os*23, respectively) nonfunctional. However, a chimeric receptor (*Os*TIR1^F-box^-*Os*COI1B^LRR^) comprising *Os*TIR1 F-box domain and *Os*COI1B LRR (version 6-*Os*23) mediated nearly 90% degradation of GFP-*Os*JAZ^23^. Nevertheless, this version probably suffered from coronatine-independent degradation, as most cells had low fluorescence levels (Supplementary Fig. 6f). Switching to *At*JAZ^23^ or extending the rice degron to 33 AAs (*Os*JAZ^33^) restored GFP levels, but attenuated the effect of coronatine (versions 6-*At*23 and 6-*Os*33, respectively). Notably, using *At*JAZ^FL^ resulted in high GFP expression and 95% coronatine-induced degradation (version 6-*At*FL), while conferring nuclear localization to GFP (Supplementary Fig. 6g), in accordance with JAZ1 localization in plants^36^, prompting us to speculate that its degron efficiency partially derives from its nuclear localization. We therefore targeted GFP-*Os*JAZ^33^ to the nucleus with an NLS (version 7) and found it to enhance both dose- and time-dependent coronatine-induced degradation, reaching >95% with 50 μM coronatine (Supplementary Fig. 6h). Thus, a chimeric *Os*TIR1^F-box^-*Os*COI1B^LRR^ receptor can effectively mediate coronatine-dependent degradation of nuclear POIs fused to an *Os*JAZ^33^ degron without evidence of coronatine-independent degradation, coronatine receptor-independent degradation or coronatine toxicity (Supplementary Fig. 6i–m). Importantly, pJAZ version 7 (henceforth pJAZ) functioned nearly as well as pRAIDRS in mediating hormone-dependent degradation of nuclear GFP (Fig. 5b) and, similar to pRAIDRS, pJAZ enabled the engineering of a molecular switch in which an endogenous protein was replaced with a coronatine-regulated exogenous protein, as demonstrated by engineering a p53 switch in human ESCs (hESCs; Fig. 5c,d).

Next, we engineered cells expressing coronatine-degradable NLS-GFP-*Os*JAZ^33^ and auxin-degradable NLS-mOrange-AID^47^ using pJAZ and pRAIDRS harbouring PuroR or BSD, respectively, and selecting these cells with puromycin and blasticidin. Flow cytometric and microscopic analyses demonstrated that pRAIDRS and pJAZ function effectively and independently in a variety of cell types, including hESCs (Fig. 5e–g), P19 mouse embryonal carcinoma cells, H1299 lung adenocarcinoma cells, HEK-293T cells, NIH/3T3 mouse embryonic fibroblasts, NCI-H358 human non-small cell lung cancer cells and HCT-116 human colorectal carcinoma cells (Supplementary Fig. 7). Importantly, both hormones induced 90–99% degradation, depending on the cell type, and did not show any cross-reactivity or interference, suggesting that neither system saturates the shared ubiquitination machinery. These data validate the applicability of pRAIDRS and pJAZ as a dual analogue molecular tuner.

### A dual molecular switch to dissect the NOTCH1 pathway

**Figure 6:**
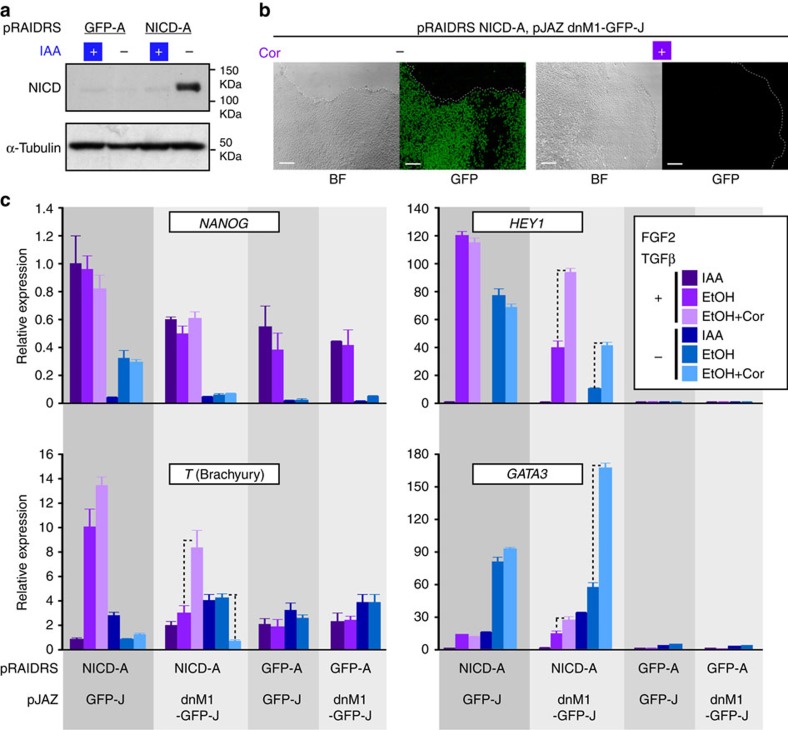
A dual switch for dissection of NOTCH1 function in hESCs. H9 hESCs were infected with pRAIDRS harbouring an shRNA targeting the full-length *NOTCH1* receptor and an NICD-AID^47^ CDS (NICD-A). As a control, cells were infected with pRAIDRS NLS-GFP-AID^47^ (GFP-A). Cells were maintained with 250 μM auxin to prevent NICD-A accumulation. Following selection and isolation of colonies with ESC morphology, cells were infected with pJAZ dnMAML1-NLS-GFP-*Os*JAZ^33^ (dnM1-GFP-J) or pJAZ NLS-GFP-*Os*JAZ^33^ (GFP-J) as control and post-selection colonies were expanded. (**a**) pRAIDRS GFP-A and NICD-A hESCs were maintained in the presence of 250 μM auxin (IAA), washed and incubated for 4 h in the presence (+) or absence (−) of auxin. A western blot analysis demonstrates NICD-A accumulation following auxin removal. α-Tubulin serves as a loading control. Experiment was repeated twice and a representative blot is displayed. (**b**) hESCs harbouring pRAIDRS NICD-A and pJAZ dnM1-GFP-J were treated with 50 μM coronatine (+) or 0.1% DMSO (−) for 1 day. Bright-field (BF) and fluorescence microscopic images demonstrate effective coronatine-dependent degradation of dnM1-GFP-J. Scale bars, 100 μm. Dashed lines mark colony borders. Experiment was conducted more than three times and representative images are displayed. (**c**) hESCs harbouring pRAIDRS NICD-A or GFP-A and pJAZ GFP-J or dnM1-GFP-J were cultured for 4 days with TeSR^TM^-E8^TM^, which contains FGF2 and TGFβ, or TeSR^TM^-E6 media, which lacks FGF2 and TGFβ, and treated with 250 μM auxin and 50 μM coronatine where indicated. Quantitative real-time PCR analysis was performed for selected genes and GAPDH-normalized values are displayed (error bars represent s.d. of three technical replicates). Dashed lines indicate instances where coronatine-mediated dnM1-GFP-J degradation restored NICD-A-dependent activity by at least twofold. Experiment was repeated three times and representative results are displayed. See also Supplementary Figs 8 and 9.

NOTCH signalling, which is inactive in undifferentiated hESCs, participates in their differentiation into embryonic lineages^37^,^38^. In mice, NOTCH was also implicated in trophectoderm formation^39^. Canonical NOTCH signalling involves ligand binding to the membrane receptor, leading to cleavage of the NOTCH intracellular domain (NICD) and its translocation to the nucleus, where it binds CSL (RBPJ) and MAML1 to activate gene transcription^40^. We sought to construct a molecular switch to dissect NOTCH1 signalling in hESCs. We infected hESCs with pRAIDRS NICD-A, which harbours an shRNA targeting the full-length *NOTCH1* receptor and an NICD-AID^47^ CDS (Supplementary Fig. 8a,b). These cells were maintained with auxin to prevent NICD-AID^47^ accumulation, which occurs quickly following auxin removal (Fig. 6a) and induces robust differentiation (Supplementary Fig. 8c,d). We then infected these cells and their pRAIDRS GFP-A control counterparts with pJAZ harbouring a dominant-negative MAML1 (ref. 38) fused to NLS-GFP and *Os*JAZ^33^ (dnMAML1-NLS-GFP-*Os*JAZ^33^, abbreviated as dnM1-GFP-J), or with pJAZ NLS-GFP-*Os*JAZ^33^ (GFP-J) as a control. Coronatine treatment effectively induced degradation of dnM1-GFP-J (Fig. 6b).

We analysed the effect of NICD-AID^47^ accumulation following auxin removal in a self-renewal condition in the presence of fibroblast growth factor 2 (FGF2) and transforming growth factor-β (TGFβ) or in their absence (differentiation condition). As depicted in Fig. 6c and Supplementary Fig. 8e, in pRAIDRS NICD-A hESCs, auxin removal led to the activation of the NOTCH targets *HEY1* and *HES5* in a manner largely independent of FGF2/TGFβ. However, the mesoderm marker *T* (Brachyury) and the ectoderm marker *SOX1* were induced by NICD-A exclusively in the presence of FGF2/TGFβ, whereas the endoderm marker *GATA6* and the trophectoderm marker *GATA3* were induced by NICD-A primarily in the absence of FGF2/TGFβ. In nearly all cases, dnM1-GFP-J hindered NICD-A-dependent transactivation and coronatine treatment attenuated the effect of dnM1-GFP-J, restoring gene expression. Moreover, *NANOG* downregulation following FGF2/TGFβ withdrawal was also NICD dependent. Taken together, these data indicate that canonical NOTCH1 signalling can induce key lineage commitment transcription factors in hESCs, and that the identity of these factors depends on FGF2/TGFβ, unveiling a cross-talk between NOTCH1 signalling and the self-renewal circuitry. In addition, the induction of *GATA3* implicates NOTCH1 in hESC trophectodermal differentiation. These data exemplify the applicability of pRAIDRS and pJAZ for the construction of dual molecular tuners capable of accurate dissection of signalling pathways in hESCs.

## Discussion

We report a molecular system that facilitates experiments that were previously unfeasible or very complicated in mammalian cells in general and ESCs in particular. Both pRAIDRS and pJAZ are easy-to-construct single vectors (Fig. 1 and Supplementary Fig. 10), which deliver all the necessary elements for the construction of rapid and reversible analogue molecular tuners or, when combined, a dual tuner.

The iterative engineering of pRAIDRS and pJAZ was aimed at enhancing their functionality in ESCs. A ‘hormone receptor/P2A/degron-fused POI/P2A/selectable marker' cassette that was codon optimized for human cells is transcriptionally driven by a PGK-1 or EF1α promoter. These promoters offer strong and stable expression in a wide variety of cells, with pPGK-1 being more stable in ESCs and pEF1α stronger^11^,^12^,^41^. The P2A peptides separating the aforementioned components are the most effective 2A peptide in mammalian cells^42^. The AID degron was minimized fivefold, to reduce interference and spontaneous cleavage. To harness the jasmonate-induced degradation pathway, we engineered a chimeric receptor, as neither *A. thaliana* nor rice coronatine receptors function in mammalian cells, and identified the minimal rice JAZ degron motif compatible with this chimeric receptor. The use of selectable markers translated in-frame with the hormone receptor and POI should ensure that drug-resistant cells are hormone sensitive. Finally, the silencing of an endogenous gene-of-interest by the pU6-driven shRNA renders each lentiviral vector an independent rescue system.

A tetracycline-based complementation approach has proven useful for gene discovery and characterization in ESCs^20^,^21^,^22^. Nevertheless, its slowness and the requirement for rtTA expression limit its use. Conversely, pAID enables rapid control of proteins, but does not offer endogenous gene inactivation, uses a large bioactive degron and is inapplicable to mammalian stem cells (Supplementary Table 1). Although auxin-dependent degradation was previously harnessed to generate molecular switches in somatic mammalian cells, this was achieved by sequential and laborious steps, such as TIR1 overexpression, POI-degron overexpression and gene-of-interest knockdown/out^13^,^14^,^15^ or, alternatively, by genomic targeting of AID degrons to both alleles of the endogenous gene combined with TIR1 overexpression^16^. Although these approaches were effective in constructing single molecular tuners, our system enables the engineering of a dual molecular tuner with unparalleled simplicity and quickness, and is particularly useful for studying ESCs, which are hard to otherwise manipulate genetically. Importantly, the rapidity of auxin-dependent protein depletion achieved with the pRAIDRS system (20–30 min for >95% degradation of NANOG and CHK1) is comparable with those reported by Han *et al*.^13^ (∼90 min), Holland *et al*.^14^ (60–100 min), Rodriguez-Bravo *et al*.^15^ (>120 min) and Lambrus *et al*.^16^ (10–30 min) in mammalian cells.

pRAIDRS and pJAZ combine the advantages of the genetic complementation and hormone-induced degradation strategies, while averting their limitations, as each vector represents a fully functional rescue system specifically tailored to mammalian stem cells and both offer rapid, reversible and titratable control of protein levels. Importantly, combining endogenous gene silencing with conditional rescue ensures high-confidence genotype-to-phenotype causal linkages. Moreover, in contrast to other conditional protein degradation/activation systems^43^,^44^,^45^,^46^, pRAIDRS and pJAZ degrons are extremely short, diminishing interference with POI localization and function. Other advantages of pRAIDRS and pJAZ are listed in Supplementary Table 1. Of note, although both pRAIDRS and pJAZ enable hormone-dependent degradation of cytoplasmic and nuclear POIs, with both systems the degradation of nuclear POIs is faster and requires lower hormone concentrations. Other noteworthy limitations of pRAIDRS/pJAZ include the following: (1) the RNAi-mediated silencing of endogenous genes, which is not always effective; (2) the constitutively active exogenous promoter driving the expression of the POI-degron fusion, which can lead to non-physiological expression levels; and (3) the lack of splice variants representation.

As a proof-of-concept, we constructed a molecular switch for the ESC master regulator NANOG. This switch enabled conditional and nearly complete rapid depletion of NANOG, recapitulating its well-established roles in mESCs^47^. By engineering a molecular switch for CHK1, we were able to elicit a series of gene-specific phenotypes as early as 45 min following hormone treatment. This degree of rapidity can facilitate the distinction between primary and secondary events, and enables high-resolution kinetic studies. Furthermore, owing to the inert and specific nature of hormone-induced degradation, we observed only minor effects following CHK1 depletion in post-selection cells, contrasting with the current conception of the role of CHK1 in normal cycling cells^27^,^28^,^48^ and in mESC self-renewal^20^. Conversely, we demonstrated that in DNA-damaged mESCs CHK1 plays a crucial protective role by restricting mitotic entry, which otherwise leads to apoptosis or differentiation. The CHK1 molecular switch represents a unique tool for screening and characterizing CHK1 inhibitors and DNA-damage sensitizers, a rapidly growing category of anti-cancer drugs^49^,^50^.

We also engineered cancer cells expressing hormone-degradable p53 and demonstrated its unleashing by auxin removal^33^, highlighting the rapid reversibility of hormone-induced degradation. Stable ectopic expression of tumour suppressors is cumbersome, as cancer cells quickly evade their effects. However, effective auxin-induced p53 degradation enabled prolonged propagation of these cells without growth inhibition or transgene silencing. We also demonstrated how pRAIDRS and pJAZ allow titratable control of protein levels, a feature that enables studies of protein dose responses and threshold levels.

By engineering a coronatine-dependent p53 switch, we demonstrated the applicability of pJAZ for rapid and simple construction of molecular switches in hESCs. Moreover, we showed how combining pRAIDRS and pJAZ yields a dual molecular switch, where auxin and coronatine control two different proteins independently. Applying this method to hESCs, we unveiled unknown aspects of the canonical NOTCH1 pathway and its integration with the hESC self-renewal network. Thus, the generation of such dual switches (or tuners) is valuable for dissecting the function of proteins and regulatory networks.

## Methods

### Cell culture

HEK-293T, HCT-116 (Obtained from S.A. Aaronson's lab at ISMMS) and NIH/393 cells were cultured in DMEM supplemented with 10% fetal bovine serum (FBS, Corning), 1 mM sodium pyruvate, 2 mM L-glutamine and PenStrep (all from Gibco). NCI-H358 and NCI-H1299 cells (obtained from the American Type Culture Collection) were cultured in RPMI-1640 (Cellgro) supplemented with 10% FBS, 1 mM sodium pyruvate, 2 mM L-glutamine and PenStrep. Validated, mycoplasma-free hESCs and mESCs were obtained from the Pluripotent Stem Cell Core Facility at ISMMS. ESCs were routinely monitored for ES-like morphology and expression of *Nanog* and Oct4 (*Pou5f1*) using quantitative real-time PCR. CCE and R1 mESCs, as well as P19 mouse embryonal carcinoma cells, were cultured in DMEM supplemented with 15% FBS, 1 mM sodium pyruvate, 2 mM L-glutamine, non-essential amino acids, PenStrep, 10 nM 2-mercaptoethanol and 100 U ml^−1^ LIF (ESGRO) on plates coated with 0.1% gelatin (Millipore, catalogue number ES-006-B). H9 hESCs were cultured with mTeSR^TM^1 (Stem Cell Technologies) on plates coated with Matrigel (BD Biosciences, catalogue number 354234). For controlling the presence of FGF2 and TGFβ, TeSR^TM^-E8^TM^ and TeSR^TM^-E6, which contain and lack FGF2/TGFβ, respectively, were used. All cells were grown at 37 °C in a humidified atmosphere of 5% CO_2_ and passaged on average twice per week. All cells were tested negative for mycoplasma using the e-Myco Mycoplasma PCR Detection Kit (iNtRON). Where indicated, cell numbers were recorded each passage and population doublings were calculated as Log_2_(cell output/cell input).

### Lentiviral infection and selection

For the production of lentiviral particles, 1 × 10^7^ HEK-293T cells were resuspended in growth media (as described above) and transfected with 20 μg lentiviral vector, 20 μg psPAX2 packaging plasmid and 10 μg pMD2.G envelope plasmid using the calcium phosphate method. Cells were then plated in a 10-cm dish and cultured for 1 day. On the second day, media were replaced and cells were incubated at 32 °C. Viral supernatants were collected on the morning and evening of the third and fourth days, passed through a 0.22- or 0.45-μm cellulose acetate filter and concentrated ∼25-fold using an Amicon Ultra-15 Centrifugal Filter (Millipore). Cells were infected with concentrated virus diluted in their appropriate media in the presence of 8 μg ml^−1^ polybrene (Sigma) for ∼16 h at 37 °C. Selection was applied 2 days following infection with either 1–2 μg ml^−1^ Puromycin (Fisher Scientific) or 10–20 μg ml^−1^ Blasticidin-S (Fisher Scientific). Where indicated, colonies (clones) of mESCs and hESCs with typical ESC morphology were manually isolated and expanded.

### Chemicals and treatments

Auxin (IAA, Fisher Scientific, catalogue number AC12216) was dissolved in ethanol to a final concentration of 500 mM. Cells were treated with 50 μM IAA or 0.01% ethanol as a control, unless otherwise indicated. Coronatine (Sigma, catalogue number C8115) was first dissolved in dimethylsulfoxide (DMSO) to a concentration of 50 mM and then diluted in DMEM to a final concentration of 5 mM. Cells were treated with 50 μM coronatine or with 0.1% DMSO as a control, unless otherwise indicated. Aphidicolin (Fisher Scientific, catalogue number AC61197) was diluted in DMSO to a final concentration of 10 mM. Cells were treated with 1 μM aphidicolin or with 0.01% DMSO as a control, unless otherwise indicated. All *trans*-retinoic acid (Fisher Scientific, catalogue number 302-79-4) was dissolved in ethanol.

### Staining

Stemgent's Alkaline Phosphatase staining kit (catalogue number 00-009) was used according to the manufacturer's protocol. Crystal violet (CV) staining was performed by incubating cells for 5 min with CV solution (10 mM CV, 10% ethanol in water), followed by three to five gentle washes with water. For both AP and CV staining, plates were scanned using a standard desktop scanner and images were digitally adjusted for brightness and contrast. Acetic acid was used to extract CV, which was then quantified using a spectrophotometer at 590 nm. DAPI (4′,6-diamidino-2-phenylindole) staining was performed by fixing cells (plated on cover slips) with 4% paraformaldehyde in PBS for 30 min, washing twice with PBS (for 5 min), treating with 0.2% Triton X-100 and 1% BSA in PBS for 30 min, washing with PBS and incubating with 0.2 μg ml^−1^ DAPI for 10 min. Cells were then washed once with PBS and mounted on microscope slides. Images acquired with a microscope were digitally adjusted for brightness and contrast. All images from the same experiment were processed identically.

### Flow cytometry

Flow cytometry was performed on a BD LSRII machine. For GFP and mOrange fluorescence analysis, cells were trypsinized, neutralized with FBS-containing media, supplemented with 0.2 μg ml^−1^ DAPI and kept on ice. Cells were gated on forward scatter area (FSC-A) and side scatter area (SSC-A), on FSC width (FSC-W) and FSC-A to eliminate cell aggregates, and on FSC-A and DAPI to eliminate dead cells. GFP and mOrange fluorescence intensities were detected using the fluorescein isothiocyanate (FITC) and DsRed channels, respectively. Background autofluorescence was measured using parental non-infected cells. Background-subtracted median fluorescence was normalized to the control-treated sample, to calculate relative median fluorescence. To calculate % degradation, relative median fluorescence was subtracted from 1. For measurement of apoptotic index, cells were collected by trypsinization together with all cells floating in the media, counted and 3 × 10^5^ cells per sample were washed twice with PBS and stained using Annexin V:PE Apoptosis Detection Kit I (BD Biosciences, catalogue number 559763) according to the manufacturer's protocol. Cells were gated on FSC-A and SSC-A, and on FSC-W and FSC-A to eliminate cell aggregates. 7-aminoactinomycin D (7-AAD) was detected using the PerCP-Cy5.5 filter. Apoptotic index was calculated as the percentage of cells that are 7-AAD negative and Annexin V-Phycoerythrin positive. For measurement of mitotic index, cells were collected by trypsinization together with all cells floating in the media, neutralized with FBS-containing media, washed and fixed by slowly adding ice-cold 70% ethanol/Hank's balanced salt solution while vortexing. Cells were kept for at least 2 h at −20 °C, washed with PBS, incubated for 15 min on ice with 0.25% Triton X-100 in PBS and resuspended in 100 μl PBS-BA (PBS supplemented with 1% BSA and 0.02% sodium azide) containing 2 μl anti-Phospho-Histone H3 Ser10 antibody (Cell Signaling, catalogue number 9706). Cells were incubated for 2 h at room temperature with gentle rocking, washed twice with PBS-BA, resuspended in 100 μl PBS-BA supplemented with Alexa Fluor 546 secondary antibody (1:200, Life Technologies), incubated for 30 min at room temperature in the dark with gentle rocking, washed with PBS-BA, resuspended in 400 μl PBS containing 50 μg ml^−1^ RNAse-A and incubated 30 min at 37 °C in the dark. Samples were then cooled, supplemented with DAPI to a final concentration of 2 μg ml^−1^ and incubated on ice for 15 min. Unstained and secondary-antibody-only samples served as controls. For analysis of cell-surface SSEA-1 expression, cells were trypsinized, washed three times with PBS supplemented with 0.5% BSA (PBSB) and 1 × 10^5^ cells were resuspended in 25 μl PBSB and 10 μl PE-conjugated anti-SSEA-1 antibody (R&D Systems, catalogue number FAB2155P) or IgG-PE for isotype control, incubated 30 min on ice, washed twice with PBSB, filtered and supplemented with DAPI to a final concentration of 0.2 μg ml^−1^.

### Quantitative real-time PCR and expression heatmaps

Total RNA was extracted using TRIZOL (Ambion) and 1–2 μg were reverse transcribed using the High Capacity Reverse Transcription Kit (Life Technologies, catalogue number 4368814) according to the manufacturer's protocol. QRT–PCR was performed in triplicates or quadruplicates using the Fast SYBR Green Master Mix (Life Technologies, catalogue number 4385612) on a LightCycler480 Real-Time PCR System (Roche). Expression was calculated using the ΔCt method. Relative expression was calculated by dividing the average level of each gene to that of the housekeeping gene *GAPDH* measured in the same cDNA sample. Gene-specific primers are listed in Supplementary Table 4. When data are displayed as bar charts, error bars represent s.d. of technical replicates. To generate gene expression heatmaps, normalized average expression levels were analysed using the Gene Cluster 3.0 software^51^. Data were log transformed and genes were mean centred. Genes were then hierarchically clustered using uncentred correlation similarity metric and average linkage.

### Western blot analysis

Cells were lysed in RIPA-B buffer (20 mM Na_2_HPO_4_ pH 7.4, 150 mM NaCl and 1% Triton X-100) supplemented with Protease Inhibitor Cocktail (Roche) for 30 min on ice with occasional vortexing, followed by 30 min centrifugation at 13,000 relative centrifugal force at 4 °C. For CDK^pY15^ detection, lysis buffer was supplemented with 1 mM dithiothreitol, 50 mM NaF, 30 mM tetrasodium pyrophosphate, 0.1 mM sodium orthovanadate, 10 mM β-glycerophosphate and 15 mM para-nitrophenylphosphate. The BCA Protein Assay Kit (Thermo Scientific, catalogue number 23225) was used to determine protein concentration. Next, 20–75 μg protein were separated by SDS–PAGE and transferred to polyvinylidene difluoride membranes (Bio-Rad). Membranes were blocked with TBST (10 mM Tris-HCl pH 7.9, 150 mM NaCl and 0.05% Tween-20) containing 3% skim milk, incubated with primary antibodies overnight, washed three times with TBST, incubated with horseradish peroxidase-conjugated secondary antibodies (Amersham), washed three times with TBST and subsequently reacted with ECL or ECL Prime (GE Healthcare). Luminescence was detected with X-ray films, which were scanned, or using the Bio-Rad ChemiDoc MP System. Blots were processed digitally by adjusting the brightness and contrast, and by rotating and cropping, when necessary. The following primary antibodies were used: rabbit anti-GFP (Invitrogen, catalogue number A-6455, 1:500), mouse anti-CHK1 (FL-393, Santa Cruz Biotechnology, catalogue number sc-8408, 1:1,000), mouse anti-β-actin (Sigma, catalogue number A2066, 1:4,000), rabbit anti-p53 (DO-1, Santa Cruz Biotechnology, catalogue number sc-6243, 1:1,000), mouse anti-p53 (Santa Cruz Biotechnology, catalogue number sc-126, 1:500), rabbit anti-p21 (Santa Cruz Biotechnology, catalogue number sc-397, 1:500), rabbit anti-NANOG (Millipore, catalogue number AB5731, 1:1,000), mouse anti-HA (Abcam, catalogue number ab16918, 1:4,000), rabbit anti-phospho Cdc2 (CDK1) Tyr15 (Cell Signaling, catalogue number 9111, 1:500), mouse anti-CDC25A (Santa Cruz Biotechnology, catalogue number sc-7389, 1:250), sheep anti-Notch-1 Intracellular Domain (R&D Systems, catalogue number AF3647, 1:200) and mouse anti-α-Tubulin (Sigma, catalogue number T9026, 1:1,500). Quantification of protein level was performed using the ImageJ software^52^. Uncropped immunoblot scans are displayed in Supplementary Fig. 9.

### mRNA-Seq

For testing the global transcriptional effect of coronatine treatment, H9 hESCs expressing pJAZ NLS-GFP-*Os*JAZ^33^ and pRAIDRS NLS-mOrange-AID^47^ were treated for 2 days with 50 μM coronatine (Cor) or 0.1% DMSO (Con). Experiment was repeated twice (replicates A and B). RNA was extracted with TRIZOL (Ambion). Sample preparation and sequencing was performed by Girihlet Inc. (www.girihlet.com). Briefly, total RNA was evaluated for quality and quantity using the Agilent RNA 6000 Nano Kit on an Agilent Bioanalyzer. Libraries were prepared using TruSeq RNA Library Prep Kit (Illumina). mRNA was isolated from 500 ng of total RNA using poly T beads and cDNA was synthesized using SuperScript Reverse Transcriptase (ThermoFisher Scientific) and random primers. The cDNA ends were blunted, ‘A' base added and adapters ligated. A total of 15 cycles of PCR were performed to generate cDNA libraries. Libraries concentration was measured using an Agilent DNA 1000 Kit on an Agilent Bioanalyzer. Libraries were sequenced on a NextSeq 500 machine (Illumina) with 1*75 bp reads.

### Data analysis

The resulting fastq files were mapped to the human genome (version hg19) using the TopHat programme (with Bowtie2). The output .bam files were processed through the Cuffquant programme to generate normalized read counts. The resulting .cxb files were processed through the Cuffdiff programme to generate fragments per kilobase of transcript per million mapped reads (FPKM) values. Raw data (fastq files), as well as FPKM values, were uploaded to the GEO database (GSE74457) and can be accessed using this link: http://www.ncbi.nlm.nih.gov/geo/query/acc.cgi?acc=GSE74457. On average, there were 7.7 × 10^7^ reads per sample, which mapped to 23,622 human genes. Lowly expressed genes with an average FPKM value <0.1 were excluded, narrowing the total gene count to 15,928. The BRB-Array Tools software^53^ was used to calculate Spearman pairwise correlation between all samples (Supplementary Fig. 5l). To identify genes that were differentially regulated following coronatine treatment (Supplementary Fig. 6m), we filtered the gene list to include genes that meet the following criteria: (1) genes that scored a *P*-value <0.05 in a two-tailed paired *t*-test comparing coronatine-treated samples with control samples; (2) genes that had a fold change >2 between coronatine and control samples in both replicates; and (3) coding genes and long non-protein-coding RNAs (excluding small RNAs). Using these criteria, only two genes demonstrated differential expression between coronatine and control samples. When the same criteria were applied to search for genes that were differentially regulated between the two biological replicates, seven such genes were identified.

### Construction of pRAIDRS and pJAZ

Initially, pRAIDRS and pJAZ vectors were synthesized by the GeneArt service as a cassette containing the following components (restriction enzyme-binding sites, REBSs, are italicized): *AscI*/pPGK-1 (partial sequence)/*SalI*/Kozak Sequence/Hormone Receptor/*EcoRV*/5′-P2A/5′-MCS/Degron/3′-MCS/3′-P2A/*NsiI*/Selectable Marker/*AatII*…*KpnI*. Cassettes were cloned using *AscI*+*KpnI* into an empty pLKO.1-Puro lentiviral vector^54^. Different versions of the vectors were then constructed by shuffling components between existing versions or adding new components using restriction enzymes. Specifically, degrons were cloned using *XmaI*+*XbaI*, hormone receptors with *SalI*+*EcoRV* and selectable markers with *PstI*+*AatII*. When indicated, restriction-free cloning (RFC)^55^ was used. Primers and shRNA sequences are listed in Supplementary Tables 2 and 3, respectively. Sequences were codon-optimized (using the GeneArt algorithm) to increase their human Codon Adaptation Index (CAI), while avoiding the generation of any REBS that would render unique REBSs in the other parts of the vector non-unique. Components were designed and constructed as follows: *Os*TIR1 is a codon-optimized (CAI=0.95): *O. sativa* (rice) *TIR1* gene (encoding NP_001052659), excluding the STOP codon. *At*COI1 is a codon-optimized (CAI=0.96) *A. thaliana COI1* gene (encoding NP_565919), excluding the STOP codon. *Os*TIR1^F-box^-*At*Coi1^LRR^: a chimeric receptor composed of an *Os*TIR1 F-box domain^4^ (AAs 1–39) and an *At*COI1 leucine-rich repeat^5^ (AAs 52–592) was constructed using RFC. Megaprimers were generated using pRAIDRS as a template and primers 1+2. These megaprimers were used with pJAZ 1 as a template, to generate pJAZ 2. *Os*COI1B is a codon-optimized (CAI=0.95) rice *COI1B* (encoding NP_001055700). *Os*TIR1^F-box^-*Os*COI1B ^LRR^: a chimeric receptor composed of a Met-HA-tagged *Os*TIR1 F-box domain^4^ (AAs 2–39) and an *Os*COI1B leucine-rich repeat^5^ (AAs 59–597) was constructed using RFC. Megaprimers were generated using pJAZ 2^HA^ as a template and primers 11+12. These megaprimers were used with pJAZ 5-*Os*23 or 5-*At*23 as a template, to generate versions 6-*Os*23 or 6-*At*23, respectively. 5′-P2A is a 2A peptide derived from porcine teschovirus-1. Codons were edited to achieve low degree (81%) of homology with the 3′-P2A sequence, to reduce recombination likelihood. 5′-MCS: four tandemly arranged 6-bp REBSs (*BstBI*, *NheI/BmtI*, *SnaBI* and *XmaI*/*SmaI*). AID^47^ is a codon-optimized (CAI=0.93) 47-AA segment that corresponds to AAs 63–109 of *A. thaliana* IAA17 (*AtIAA17*, NP_171921). AID^33^ (corresponding to *AtIAA17* AAs 63–95) was generated by PCR amplification using AID^47^ as a template and primers containing REBSs enabling replacement of the degron segment. *At*JAZ^23^ is a codon-optimized (CAI=0.96) 23-AA segment that corresponds to AAs 199–221 of *A. thaliana* JAZ1 (*At*JAZ1, NP_973862). *At*JAZ^31^ is an extended version of *At*JAZ^23^ and was PCR-amplified with *At*JAZ^23^ as a template and primers 3+4, and then cloned with *XmaI*+*XbaI*. *At*JAZ^FL^ is the full-length *A. thaliana* JAZ1 protein (non-codon optimized) and was PCR-amplified from a JAZ1-containing plasmid^5^ with primers 5+6, and cloned with *XmaI*+*XbaI*. *Os*JAZ^23^ is a codon-optimized (CAI=0.98) 23-AA segment corresponding to AAs 114–136 of *O.sativa* JAZ1 (*Os*JAZ1, NP_001064513). *Os*JAZ^33^ is an extended version of *Os*JAZ^23^ (corresponding to *Os*JAZ1 AAs 109–141) and was PCR amplified with *Os*JAZ^23^ as a template and primers 7+8, and cloned with *XmaI*+*XbaI*. 3′-MCS: four tandemly arranged 6-bp REBSs (*XbaI*, *HpaI*, *BamHI* and *PstI*). It is noteworthy that *PstI* is not unique in vectors containing pEF1α or *Os*COI1B^LRR^. 3′-P2A is identical to 5′-P2A, except for different codon usage. PuroR is a codon-optimized (CAI=0.92) N-acetyltransferase gene. BSD is a codon-optimized (CAI=0.96) Blasticidin-S deaminase gene. HA tag: to generate pRAIDRS 7^HA^, an HA tag (YPYDVPDYA), preceded by a methionine (Met), was inserted upstream of *Os*TIR1 by cassette PCR amplification with pRAIDRS as the template and primers 9+10, and cloning this cassette into pRAIDRS with *SalI*+*BstBI*. To generate pJAZ 2^HA^, a Met-preceded HA tag was cloned upstream of the *Os*TIR1^F-box^-*At*COI1^LRR^ by cassette PCR amplification with pJAZ 2 as the template and primers 9+10, and cloning this cassette into pJAZ with *SalI*+*BstBI*. GFP: enhanced GFP was PCR amplified from pLKO.1-Puro-IRES-GFP with primers 16+14 and cloned into pJAZ or pRAIDRS using *NheI+XmaI*. mOrange was PCR amplified from pFUW-mOrange with primers 17+15 and cloned into pJAZ or pRAIDRS with *NheI+XmaI*. NLS: an SV40 large T-antigen NLS (PKKKRKV) was fused to the amino terminus of GFP or mOrange by PCR amplifying an NLS-GFP cassette with primers 13+14 or an NLS-mOrange cassette with primers 13+15, and cloning into pJAZ or pRAIDRS with *NheI+XmaI*. *Hs*SKP2^F-box^-*At*COI1^LRR^: a chimeric receptor composed of an *Homo sapiens* SKP2 (*Hs*SKP2) F-box domain (AAs 95–132) and *At*COI1 leucine-rich repeat^5^ (AAs 50–592) was constructed using RFC. Megaprimers were generated using *Hs*SKP2-containing plasmid^56^ as a template and primers 18+19. These megaprimers were used with pJAZ 1 as a template to generate pJAZ 3. *Hs*SKP1-*At*COI1^LRR^ fusions: chimeric receptors composed of either full-length *Hs*SKP1 or an N-terminal-truncated *Hs*SKP1 lacking AAs 1–129 (*Hs*SKP1^Δ1-129^) and either *At*TIR1^F-box^-*At*COI1^LRR^ or just *At*Coi1^LRR^ were constructed using RFC. Megaprimers were generated using pCDNA3.1-SKP1-HA as a template and the following primer combinations: 20+21 for *Hs*SKP1-*Os*TIR1^F-box^-*At*COI1^LRR^, 20+22 for *Hs*SKP1-*At*COI1^LRR^, 20+23 for *Hs*SKP1^Δ1-129^-*Os*TIR1^F-box^-*At*COI1^LRR^ and 20+24 for *Hs*SKP1^Δ1-129^-*At*COI1^LRR^. Megaprimers were used with pJAZ 2 as a template to generate pJAZ 4a-d. pEF1α: human EF1α promoter was cloned using RFC: megaprimers were generated using pEF1α-BirA-V5-His as a template and primers 25+26. These megaprimers were used to switch pPGK-1 into pEF1α in pRAIDRS and pJAZ. Site-directed mutagenesis^57^ was performed with primers 33+34 to eliminate the *AgeI* site in pEF1α.

### Construction of rescue systems

In general, rescue system vectors were constructed using the two-step cloning protocol (Supplementary Fig. 10). The specific components used were as follows: for pRAIDRS AID^47^-NANOG (A-NANOG): an shRNA cassette targeting mouse *Nanog* 3′-UTR was generated by annealing oligonucleotides 101+102, as previously described^58^, and cloning into pRAIDRS with *AgeI*+*EcoRI*. Mouse *Nanog* CDS was amplified from pCR4-Nanog using primers 27+28 and cloned into the shRNA-containing pRAIDRS with *XbaI*+*BamHI*. For pRAIDRS p53-AID^47^ (p53-A): mouse p53 CDS was amplified from pSIN-EF2-Myc-Trp53 using primers 29+30 and cloned into pRAIDRS with *BstBI*+*NheI*. For pRAIDRS AID^47^-CHK1 (A-CHK1): an shRNA cassette targeting mouse *Chk1* 3′-UTR was generated by annealing oligonucleotides 103+104 and cloning into pRAIDRS with *AgeI*+*EcoRI*. Mouse *Chk1* CDS was amplified from pGEM-T-Chk1 using primers 31+32 and cloned into pRAIDRS with *XbaI*+*BamHI*. For pRAIDRS NICD-AID^47^ (NICD-A): an shRNA cassette targeting human *NOTCH1* 3′-UTR was generated by annealing oligonucleotides 105+106 and cloning into pRAIDRS with *AgeI*+*EcoRI*. Human NICD CDS was amplified from EF.hICN1.Ubc.GFP (Addgene Plasmid 17626) using primers 37+38 and cloned into pRAIDRS with *NheI*+*SnaBI*. For pJAZ *Os*JAZ^33^-p53 (J-p53): an shRNA cassette targeting human *TP53* 3′-UTR was generated by annealing oligonucleotides 109+110 and cloning into pLKO.1 Puro with *AgeI*+*EcoRI*. Next, the cassette was transferred to pJAZ with *EcoRI*+*SphI*. Human p53 CDS was amplified from pLenti6/V5-p53_wt p53 (Addgene Plasmid 22945) using primers 41+42 and cloned into pJAZ with *XbaI*+*BamHI*. For pJAZ dnMAML1-NLS-GFP-*Os*JAZ^33^ (dnM1-GFP-J): dominant-negative human MAML1 (dnMAML1)^38^, corresponding to MAML1 AAs 13–74, was cloned from pHAGE-N-V5-MAML1-FL (Addgene Plasmid 37048) using primers 39+40 and cloned into pJAZ NLS-GFP-*Os*JAZ^33^ with *BstBI*+*NheI*.

### Additional plasmids

psPAX2 second-generation packaging plasmid and pMD2.G envelope plasmid were purchased from Addgene. pLKO.1-Puro-IRES-mCherry, pGEM-T-Chk1 and pSIN-EF2-Myc-Trp53 were kindly provided by Dr Dung-Fang Lee. pFUW-mOrange was kindly provided by Dr Carlos-Filipe Pereira. pCDNA3.1-Skp1-HA plasmid was kindly provided by Dr Doris Germain. pCR4-Nanog and pEF1α-BirA-V5-His were kindly provided by Dr Christoph Schaniel.

### Data availability

All relevant data, including full plasmid sequences, are available from the authors on request. mRNA sequencing data generated during this study were deposited in NCBI Gene Expression Omnibus (GEO) database (http://www.ncbi.nlm.nih.gov/geo/) as series GSE74457 and samples GSM1921000–GSM1921003.

## Additional information

**How to cite this article:** Brosh, R. *et al*. A dual molecular analog tuner for dissecting protein function in mammalian cells. *Nat. Commun.* 7:11742 doi: 10.1038/ncomms11742 (2016).

## Supplementary Material

Supplementary Information Supplementary Figures 1 - 10, Supplementary Tables 1 - 4 and Supplementary References
