# Supplementary material for: A dual molecular analogue tuner for dissecting protein function in mammalian cells
Source: Nat Commun. 2016 May 27;7:11742. doi: 10.1038/ncomms11742 (PMC4895048; doi:10.1038/ncomms11742)
Supplement: Supplementary Information — Supplementary Figures 1 - 10, Supplementary Tables 1 - 4 and Supplementary References [file ncomms11742-s1.pdf]

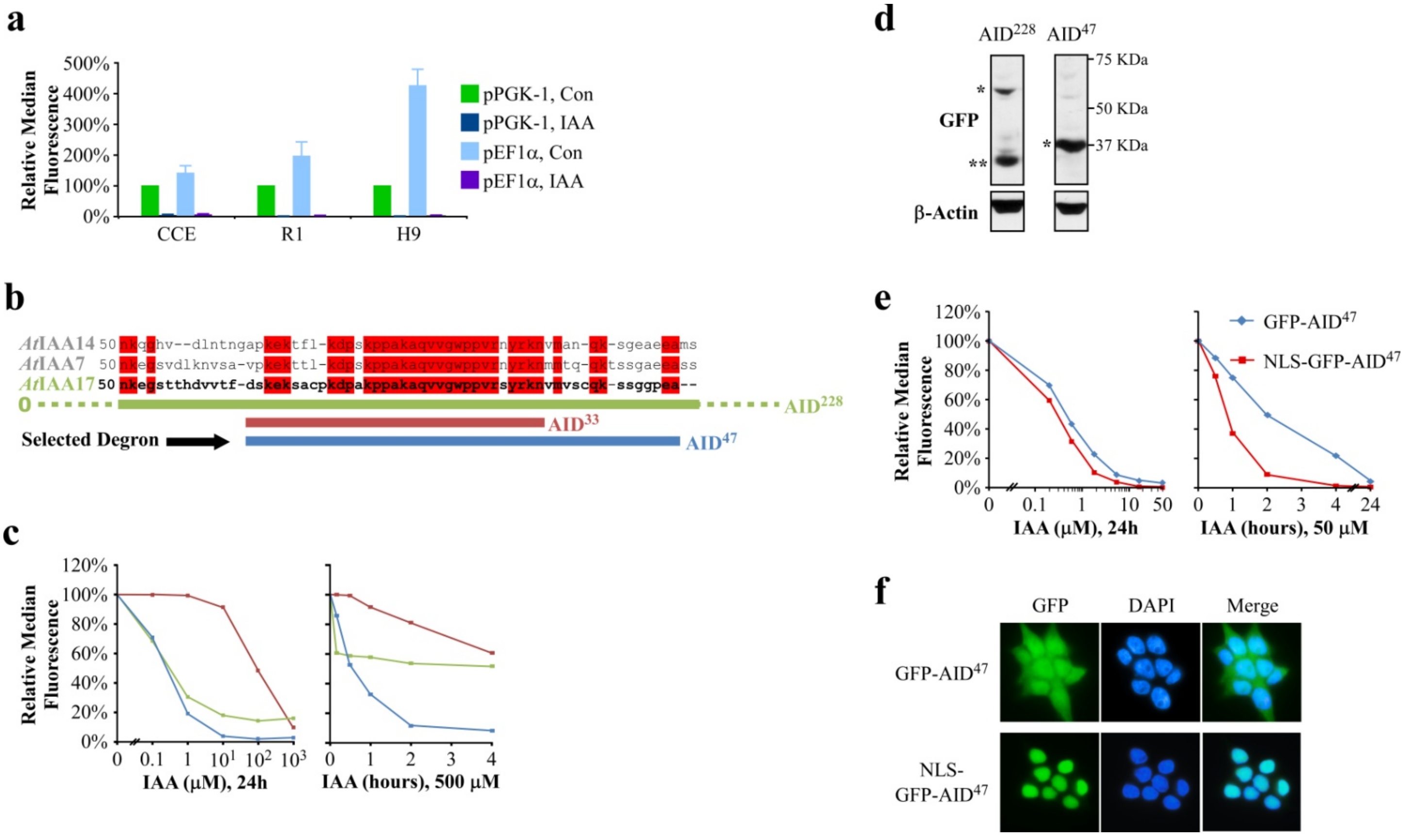

Supplementary Figure 1. Optimization and Characterization of pRAIDRS. **(a)** pRAIDRS harboring either a PGK-1 or an EF1α promoter and GFP-AID^{47} was infected into CCE and R1 mESCs or H9 hESCs. Cells were selected, treated with 0.01% EtOH (Con) or 50 μM auxin (IAA) and analyzed for GFP fluorescence by flow cytometry. For each cell type, values were normalized to the "pPGK-1, Con" sample. Data represent averages and s.d. of 2 biological replicates. (**b**) A conserved region among three *A. thaliana* auxin-degradable proteins (*At*IAA14, NP_193191; *At*IAA7, NP_188945; *At*IAA17, NP_171921)[1] and three versions of *At*IAA17 that were tested as degrons in pRAIDRS. Conserved residues are highlighted in red. Superscript numbers in degron names indicate length in AAs. (**c**) Auxin-induced degradation mediated by different AID degrons. HEK-293T cells were infected with pRAIDRS vectors harboring a GFP fused to degrons of different sizes. Cells were treated with increasing concentrations of IAA and analyzed 24 hours later (left panel), or with 500 μM IAA for the indicated time periods (right panel). GFP fluorescence was analyzed by flow cytometry. Data series are color-coded as in (b). Experiment was conducted twice and representative results are displayed. (**d**) A WB analysis of GFP in pRAIDRS-infected HEK-293T cells. Untreated cells are expressing GFP fused to either the full-length *At*IAA17 (AID^{228}) or to AID^{47}. The bands corresponding to GFP-AID^{228} and GFP-AID^{47} are marked with a single asterisk at an expected molecular weight of 58 and 36 kDa, respectively. An additional band (two asterisks) corresponds to cleaved (unfused) GFP protein with the expected molecular weight of 27 kDa. Experiment was conducted 2 times and representative results are displayed. Note the complete absence of cleaved GFP in the right lane, indicating that this spontaneous cleavage occurs only with AID^{228}. This phenomenon can explain the biphasic kinetics of GFP-AID^{228} degradation (panel b) whereas the fused GFP is rapidly degraded, while the unfused is insensitive to auxin. (**e**) The effect of nuclear localization on auxin-induced degradation of GFP-AID^{47}. HEK-293T cells were infected with pRAIDRS containing either GFP-AID^{47} or NLS-GFP-AID^{47}. Cells were treated with increasing concentrations of IAA and analyzed after 24 hours (left panel), or with 50 μM IAA for the indicated time periods (right panel). GFP fluorescence was analyzed by flow cytometry. Experiment was conducted 3 times and representative results are displayed. (**f**) GFP and DAPI microscopic images of HEK-293T cells infected pRAIDRS containing either GFP-AID^{47} or NLS-GFP-AID^{47}.

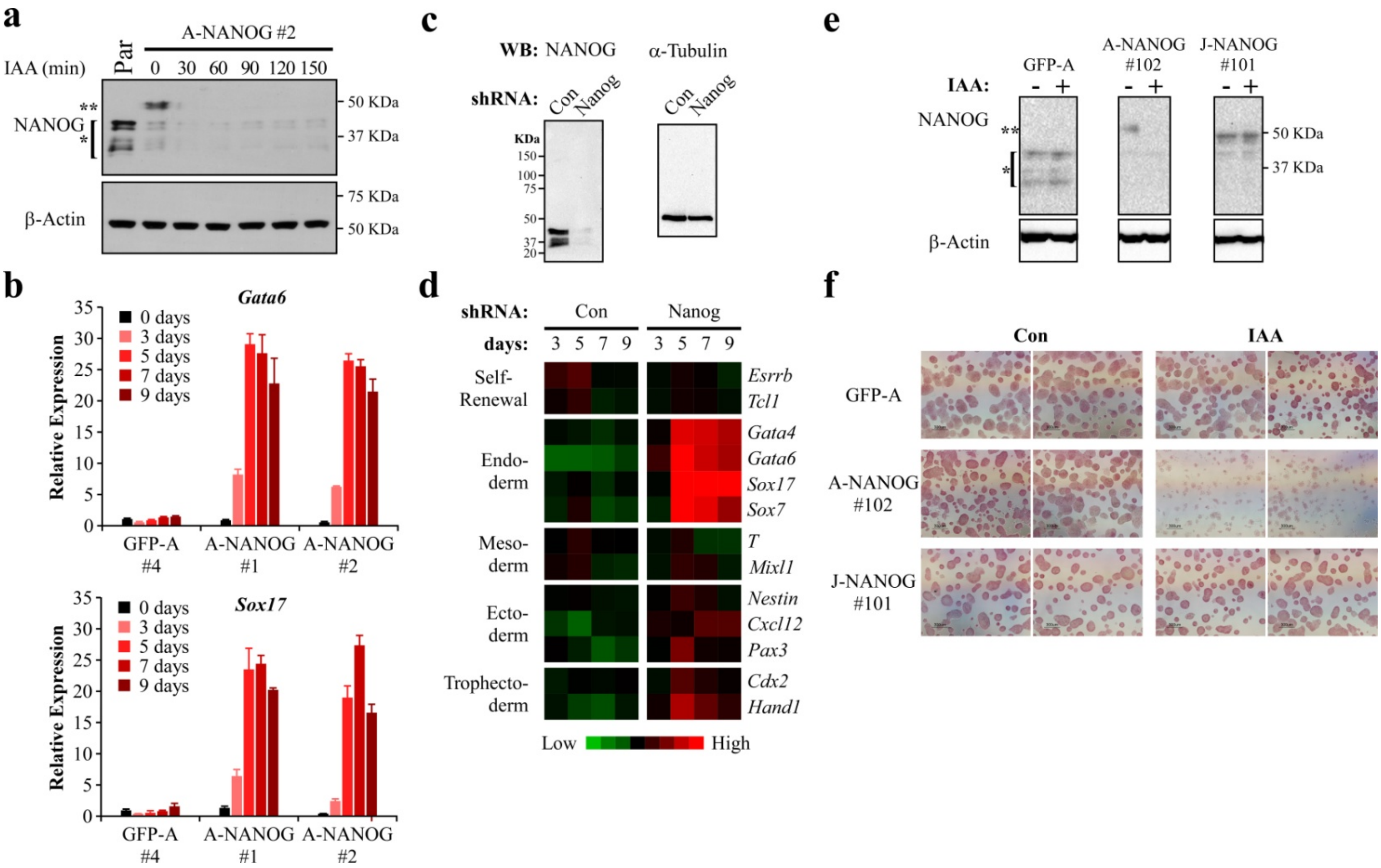

Supplementary Figure 2. An Auxin-Degradable NANOG Rescue System in mESCs. (**a**) A-NANOG #2 mESCs were treated with 50 μM auxin (IAA) for the indicated time periods. A WB analysis demonstrates complete depletion of A-NANOG following 30 minutes of auxin treatment. Endogenous NANOG and exogenous A-NANOG are marked by * and **, respectively. Experiment was conducted 3 times and representative results are displayed. (**b**) Selected mRNA expression patterns from the heatmap in Figure 2d displayed as bar charts. Error bars represent s.d of three technical replicates. (**c-d**) shRNA-mediated knockdown of NANOG in CCE mESCs. Cells were infected with pLKO.1-Puro-IRES-mCherry harboring either a Luciferase shRNA (sh-Con) or a *Nanog* shRNA (sh-Nanog). Two days post infection cells were selected with 1 μg/ml Puromycin for the indicated number of days. On the 3rd day of selection cells were collected for the first QRT-PCR analysis time point and the rest of the cells were replated for the following time points. For WB analysis (c), cells were collected on the 5th day of selection. QRT-PCR data are presented as a heatmap (d). Experiment was conducted twice and representative results are displayed. (**e**) mESCs were infected with either pRAIDRS GFP-AID[47] (GFP-A), pRAIDRS AID[47]-NANOG (A-NANOG) or pRAIDRS *Os*JAZ[33]-NANOG (J-NANOG), which harbors a *Nanog* shRNA and a *Nanog* coding sequence fused to the *Os*JAZ[33] degron. In the latter cells endogenous *Nanog* is replaced by an exogenous *Nanog* that does not contain an AID degron and, therefore, should be auxin resistant. A WB analysis depicts endogenous NANOG (*) and exogenous degron-fused NANOG (**) in a pool of GFP-A mESCs and clones of A-NANOG and J-NANOG mESCs. Experiment was conducted once. (**f**) GFP-A (pool) and clones of A-NANOG and J-NANOG mESCs were plated at low density, grown in the presence of EtOH (Con) or auxin (IAA) for 4 days and assayed for alkaline phosphatase activity. Low magnification pictures were taken using a bright-field microscope. Note that only A-NANOG mESCs demonstrated reduction in AP-positive colony number upon auxin treatment.

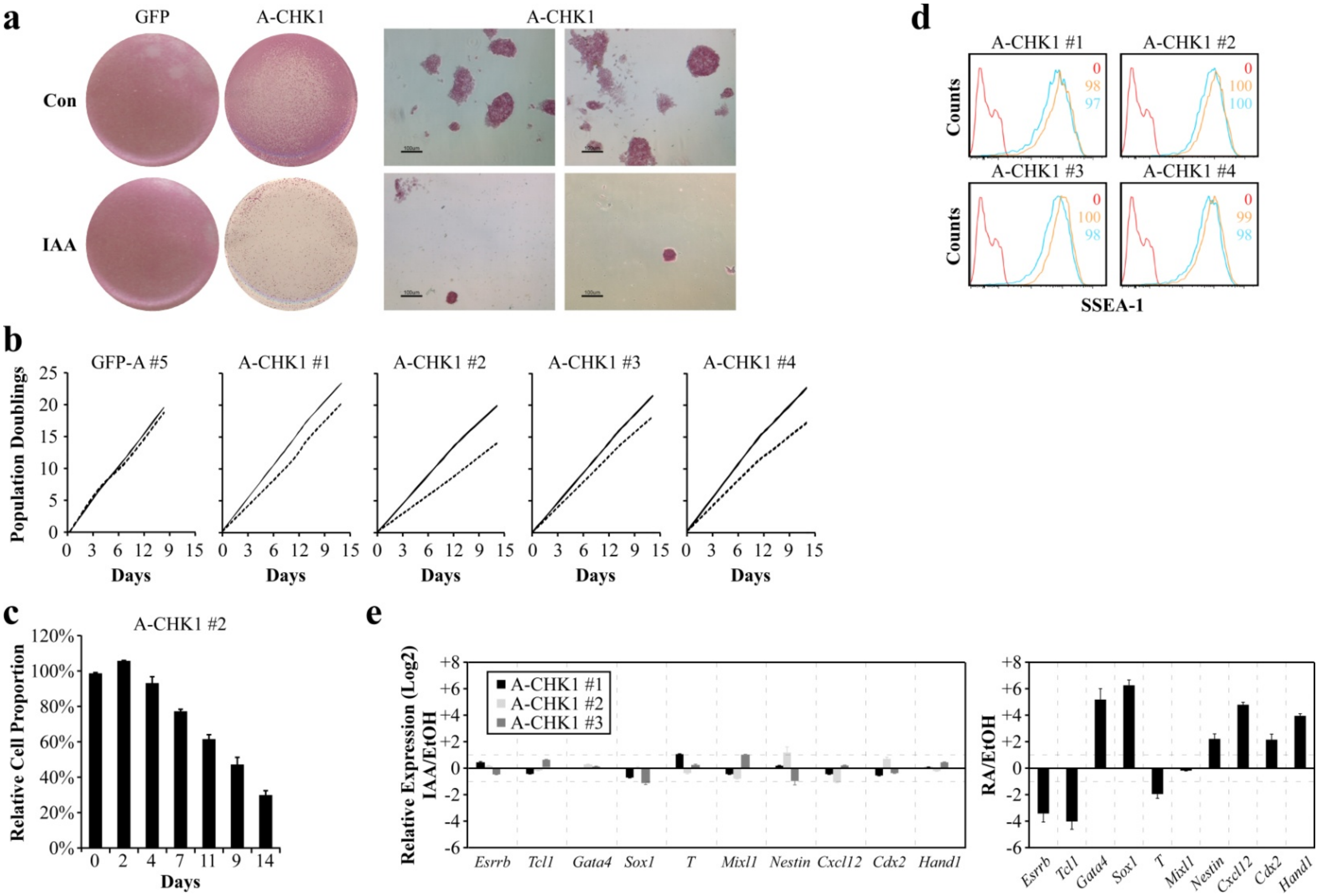

Supplementary Figure 3. CHK1 Depletion under Normal Growth Conditions in mESCs. (**a**) CCE mESCs were infected with pRAIDRS GFP-A or A-CHK1 and selected in the presence of EtOH (Con) or auxin (IAA). After colonies emerged, cells were assayed for alkaline phosphatase activity. Left, low magnification scans. Right, bright-field microscope images. Scale bars, 100 µm. Experiment was conducted 3 times and representative results are displayed. (**b**) Following selection, the indicated clones were cultured in the presence of 0.01% EtOH (solid lines) or 50 µM IAA (dashed lines). Cells were counted and replated every 3-4 days and population doublings were calculated as Log_2(cell output/cell input). Experiment was conducted once for these clones. (**c**) CCE A-CHK1 clone #2 cells were subjected to a competition assay[2]. Cells were labeled with mCherry fluorescence protein and co-cultured with control GFP-A CCE cells in the presence of 0.01% EtOH or 50 µM IAA. Cells were collected every 2-3 days and assayed for GFP and mCherry fluorescence by flow cytometry. The percentage of A-CHK1 cells was calculated for each time point and the values for the auxin-treated cells were normalized to those of EtOH-treated cells. An average growth rate decrease of 8% per day was calculated for auxin-treated A-CHK1 cells compared to the EtOH-treated controls. For comparison, a similar assay performed by Ivanova *et al*. reported drastic reduction in cell proportion following knock-down of genes involved in mESC self-renewal[3]. Experiment was conducted twice and representative results are displayed. (**d**) The indicated clones were treated with 0.01% EtOH (orange histograms) or 50 µM IAA (blue histograms) for 2 days and analyzed for cell-surface pluripotency marker SSEA-1 expression by flow cytometry. Red histogram, isotype control. Numbers indicate percentages of SSEA-1 positive cells and are color-coded similarly to the histograms. Experiment was conducted once. (**e**) The indicated clones were treated with 0.01% EtOH or 50 µM auxin (IAA) for 3 days and analyzed for the expression of self-renewal and differentiation markers by QRT-PCR. Bars represent Log_2(fold change[IAA/EtOH]). Error bars represent s.d of 3 technical replicates. Auxin-induced differentiation experiment was performed 3 times and representative results are displayed. On the right, data from retinoic acid (RA, 5 µM, 3 days) and EtOH (Con, 0.05%, 3 days) treated mESCs serve as a positive control for mESC differentiation transcriptional alterations.

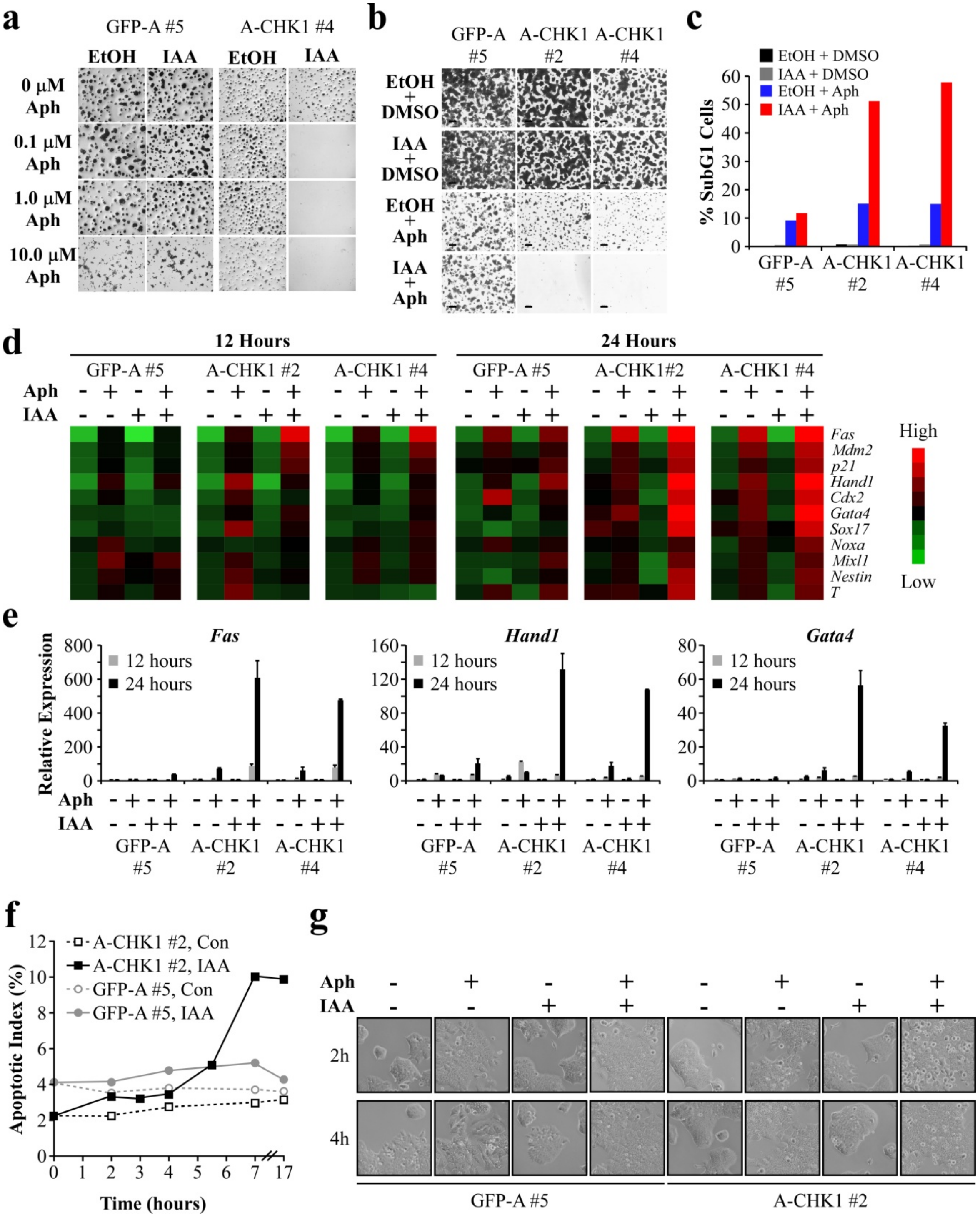

Supplementary Figure 4. CHK1 Protects mESCs from Aphidicolin-Induced Death and Differentiation. CCE mESCs were infected with pRAIDRS A-CHK1 or GFP-A. Selected clones were analyzed for their CHK1-dependent response to aphidicolin treatment. (**a**) The indicated clones were pre-treated with 0.01% EtOH or 50 μM auxin (IAA) for one day. Cells were then treated with the indicated concentrations of aphidicolin (Aph). Equal concentrations of DMSO were applied in all conditions. The next day, cells were stained with crystal violet and plates were scanned. (**b**) The indicated clones were pre-treated with 0.01% EtOH or 50 μM auxin (IAA) for one day. Cells were then treated with 1 μM aphidicolin (Aph) or 0.01% DMSO for 12 hours, trypsinized, resuspended in fresh media and 5% of the total volume was replated. Replated cells were grown in the absence of aphidicolin for two days, stained with crystal violet and microscope images were acquired. Scale bars, 200 μm. Experiment was conducted 2 times and representative results are displayed. (**c**) The indicated mESC clones were pre-treated with EtOH or auxin (IAA) for one day. Cells were then treated with 1 μM

aphidicolin (Aph) or 0.001% DMSO for 12 hours and analyzed for DNA content using propidium iodide (PI). The percentage of cells with less than 2N DNA content (% SubG1 cells) is plotted. Experiment was repeated 2 times and representative results are displayed. (**d-e**) CHK1 depletion in aphidicolin-treated cells leads to induction of p53 transcriptional targets and differentiation markers. The indicated mESC clones were pre-treated with 0.01% EtOH or 50 μM IAA for one day and were then treated with 1 μM aphidicolin (Aph) or 0.01% DMSO for 12 or 24 hours. QRT-PCR analysis was performed for selected markers, including p53 target genes (*Fas*, *Mdm2*, *p21*, *Noxa*), endodermal differentiation markers (*Gata4*, *Sox17*), mesodermal differentiation markers (*T*, *Mixl1*), ectodermal differentiation marker (*Nestin*) and trophectodermal differentiation markers (*Cdx2*, *Hand1*). Normalized average expression levels are represented as a heatmap (d) and the expression levels of selected genes are also displayed as bar charts (e). Experiment was repeated 3 times and representative results are displayed. (**f**) The indicated clones were pre-treated with 1 μM aphidicolin for one day and were then treated with EtOH (Con) or auxin (IAA) for the indicated time periods. Apoptotic index was calculated as the percentage of Annexin V-positive, 7-AAD-negative cells. Experiment was repeated twice and representative results are displayed. (**g**) Cells were treated as described above. Bright-field microscope images showing synchronous cell rounding, a feature of late mitotic cells, two hours following auxin treatment in aphidicolin-treated A-CHK1#2 cells, but not in GFP-A #5 cells. Experiment was performed more than 3 times and representative results are displayed.

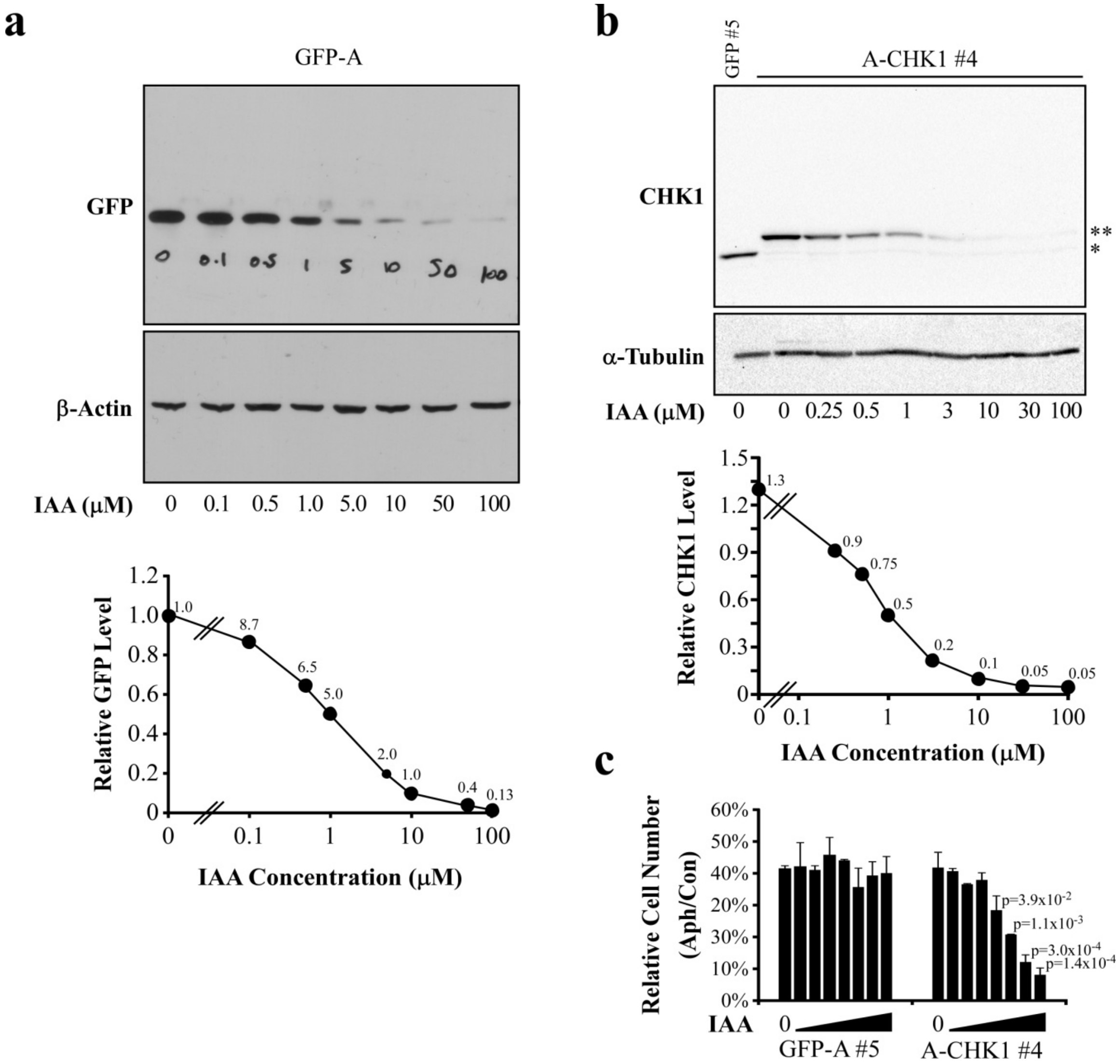

Supplementary Figure 5. pRAIDRS Enables Titratable Regulation of Protein Level. (**a**) HEK-293T cells infected with pRAIDRS GFP-AID[47] were treated with the indicated concentrations of IAA for one day. Protein levels were analyzed by A WB. β-Actin serves as a loading control. Protein levels were quantified and normalized levels are presented in the chart below. Experiment was performed 3 times and representative results are displayed. (**b**) CCE mESCs clones GFP-A #5 and A-CHK1 #4 were treated with the indicated concentrations of IAA for one day. Protein levels were analyzed by A WB (top). α-Tubulin served as a loading control. Protein levels were quantified and relative levels of total CHK1 (calculated as the level of endogenous CHK1 (*) plus the level of A-CHK1(**) divided by the level of α-Tubulin and normalized so that endogenous CHK1 in GFP #5 was set to 1) are plotted (bottom). (**c**) Cells were treated with increasing concentrations of auxin (as in panel b). The next day, media was supplemented with 1 μM aphidicolin (Aph) or 0.01% DMSO (Con) for 24 hours. Cells were stained with crystal violet, washed, and the remaining crystal violet was extracted with acetic acid and quantified using a spectrophotometer at 590 nm. Relative cell number was calculated as the ratios of Aph/Con–treated samples. Error bars represent s.d of 3 technical replicates. Statistical significance was calculated using a non-paired t-test for each IAA concentration compared with concentration 0 μM and statistically significant p-values (<0.05) are provided next to their corresponding bars. Experiment was performed twice and representative results are displayed.

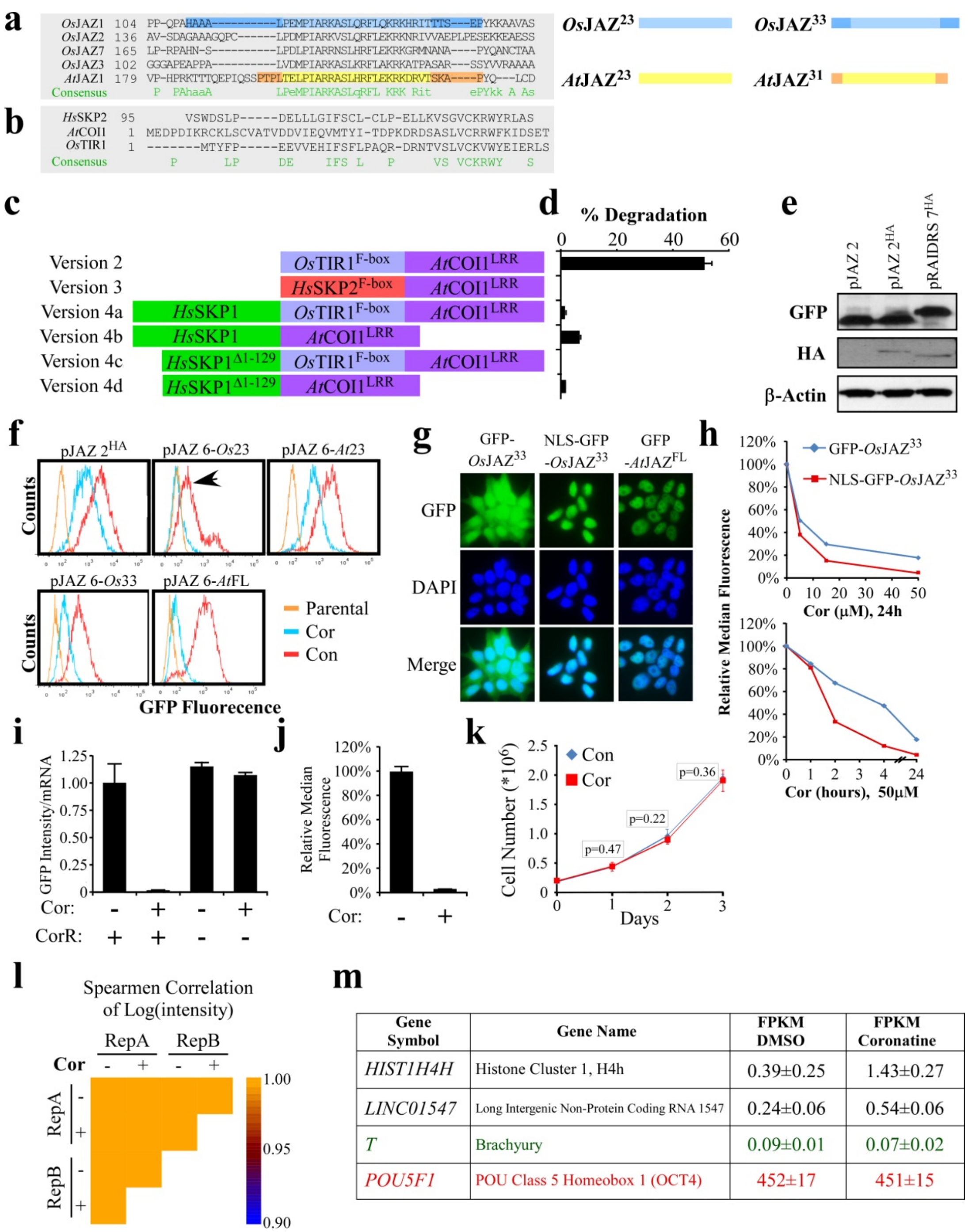

Supplementary Figure 6. Optimization and Characterization of pJAZ. (**a**) Sequence alignment of *O. sativa* coronatine-degradable proteins[4] *Os*JAZ1 (NP_001064513), *Os*JAZ3 (NP_001049167), *Os*JAZ7 (NP_001063273), *Os*JAZ3 (NP_001049166) and *A. thaliana At*JAZ1 (NP_564075). Degron sequences used in this study are highlighted and degron names indicated on the right. (**b**) Sequence alignment of the F-box domains used in this study: *hs*SKP2 (NP_005974) AAs 95-132, *At*COI1 (NP_565919) AAs 1-51 and *Os*TIR1 (NP_001052659) AAs 1-39. (**c**) Schematic representation of the hormone receptor structure of different pJAZ versions. Each receptor is composed of combinations of: human SKP2 F-Box domain (*Hs*SKP2$^{\text{F-box}}$), either full-length human SKP1 (*Hs*SKP1) or *Hs*SKP1 lacking its F-box binding region (AA 1-129, *Hs*SKP1$^{\Delta 1\text{-}129}$), rice TIR1 F-box domain (*Os*TIR1$^{\text{F-box}}$) and *A. thaliana* COI1 Leucine-Rich Repeats (*At*COI1^{LRR}). (**d**) HEK-293T cells were infected with the pJAZ versions depicted in (c). All pJAZ vectors harbored GFP-*At*JAZ^{23}. Cells were treated with 0.1% DMSO or 50 μM coronatine for 24 hours. GFP level was analyzed by flow cytometry and % Degradation was calculated as described in Methods. Experiment was conducted twice and error bars represent s.d. of biological replicates. (**e**) A WB analysis of HEK-293T cells infected with pJAZ 2 (harboring *Os*TIR1$^{\text{F-box}}$-*At*COI1^{LRR} and

GFP-*At*JAZ[23]), pJAZ 2^{HA} (harboring HA-*Os*TIR1$^{F\text{-}box}$-*At*COI1^{LRR} and GFP-*At*JAZ[23]) or pRAIDRS 7^{HA} (harboring HA-*Os*TIR1 and GFP-AID[47]). β-Actin serves as loading control. Experiment was conducted twice and representative results are displayed. See Supplementary Figure 9 for un-cropped blots. (**f**) GFP fluorescence histograms derived from flow cytometric analysis of HEK-293T cells that were infected with the indicated pJAZ versions, selected and treated with either 0.1% DMSO (Con) or 50 μM coronatine (Cor). Parental HEK-293T cells were analyzed as a control for background autofluorescence. An arrow points to a population of GFP^{low} cells in control-treated cells infected with pJAZ 6-*Os*23. The top three and bottom two panels derive from different experiments. (**g**) GFP and DAPI microscopic images of HEK-293T cells infected with pJAZ 6 harboring GFP fused to the indicated degrons. (**h**) HEK-293T cells were infected with pJAZ 7 harboring either GFP-*Os*JAZ[33] or NLS-GFP-*Os*JAZ[33]. Cells were selected and treated with increasing concentrations of coronatine (Cor) and analyzed after 24 hours (left panel), or with 50 μM coronatine for the indicated time periods (right panel). GFP fluorescence was analyzed by flow cytometry. Experiment was conducted twice and representative results are displayed. (**i**) NLS-GFP-*Os*JAZ[33] degradation is dependent on the presence of coronatine and coronatine receptor. HEK-293T cells were infected in duplicates with pJAZ (version 7) containing an NLS-GFP-*Os*JAZ[33] or with the same vector lacking coronatine receptor (CorR). To control for the structure and size of the vector, CorR (*Os*TIR1$^{F\text{-}box}$-*Os*COI1B^{LRR}) was replaced with an auxin receptor (*Os*TIR1). Cells were selected and treated with 50 μM coronatine (Cor) or 0.1% DMSO. GFP fluorescence was measured using a flow cytometer and was normalized to the relative level of GFP mRNA in each sample in order to control for differences in vector copy number and expression levels. Notably, the normalized fluorescence level of GFP was not affected by the presence of CorR in the absence of coronatine, indicating lack of coronatine-independent degradation. Moreover, coronatine treatment led to GFP degradation in CorR-dependent manner. Error bars represent s.d. of two biological replicates. (**j-k**) Coronatine treatment does not affect proliferation in human ESCs. H9 mESCs expressing pJAZ NLS-GFP-*Os*JAZ[33] were grown in the presence 50 μM coronatine (Cor) or 0.1% DMSO (Con). Flow cytometry was used to validate coronatine-dependent GFP degradation after 1 day of treatment (j). Cells were counted daily and growth curves are plotted in panel k. Experiment was performed in triplicates and a two-tailed paired t-test was used to calculate statistical significance. p-values are presented for each time point. Error bars represent s.d of technical replicates. (**l-m**) Coronatine treatment does not affect global gene expression patterns in human ESCs. H9 mESCs expressing pJAZ NLS-GFP-*Os*JAZ[33] were treated for 2 days with 50 μM coronatine or 0.1% DMSO and collected for mRNA-Seq analysis. Experiment was performed twice (RepA and RepB). Detailed description of the mRNA-Seq and data analysis is provided in Methods. Pairwise Spearman analysis shows perfect correlation between all samples (l). Using the criteria explained in Methods, only two genes (*HIST1H4H* and *LINC01547*) demonstrated differential expression between coronatine and control samples (m). As controls, FPKM values (Fragments Per Kilobase of transcript per Million mapped reads) are listed for Brachyury (*T*) and OCT4 (*POU5F1*), representing a differentiation marker (not expressed in hESCs) and a pluripotency marker (highly expressed in hESCS), respectively. These data suggest that *HIST1H4H* and *LINC01547* are expressed at very low levels, and are unlikely significant to hESCs biology. Moreover, when the same search criteria were applied to identify genes that are differentially regulated between RepA and RepB (regardless of coronatine treatment), 7 genes were identified (data not shown). This suggests that the two genes differentially expressed following coronatine treatment do not represent a significant transcriptional response, and are likely a result of inherent experimental noise.

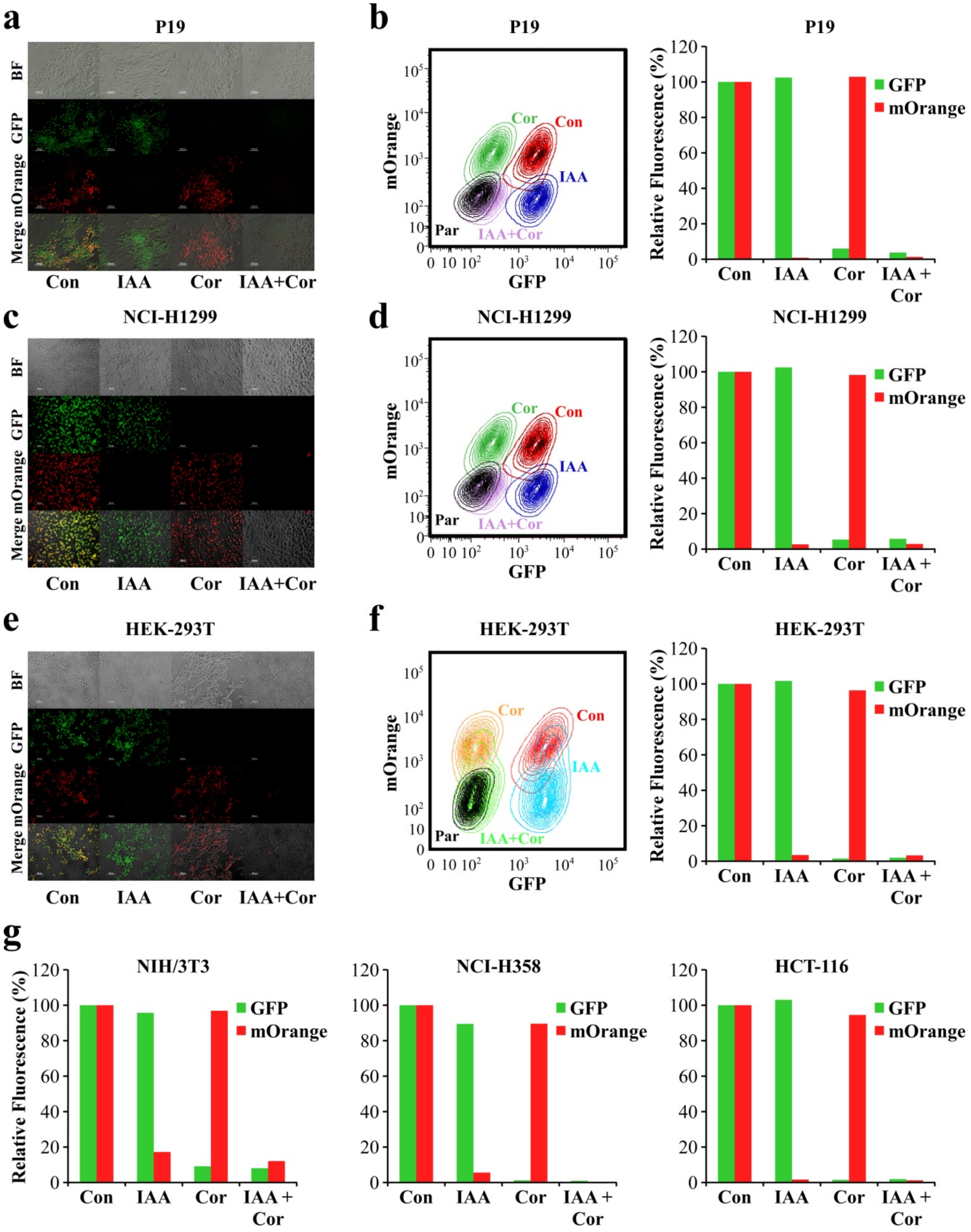

Supplementary Figure 7. pRAIDRS and pJAZ Function Independently and Simultaneously in Multiple Cell Types. P19 mouse embryonal carcinoma cells (**a-b**), NCI-H1299 human lung adenocarcinoma cells (**c-d**) and HEK-293T human embryonic kidney cells (**e-f**) were infected with pJAZ NLS-GFP-*Os*JAZ[33] (harboring PuroR) and pRAIDRS NLS-mOrange-AID[47] (harboring BSD) and selected with puromycin and blasticidin. Cells were treated with either EtOH and DMSO (Con), auxin and DMSO (IAA), EtOH and coronatine (Cor) or auxin and coronatine (IAA+Cor). After 24 hours, bright-field (BF) and fluorescence microscope images were taken (a, c and e) and cells were subjected to flow cytometric fluorescence analysis (b, d and f, contour plots on the left; quantification on the right). Parental non-infected cells (Par) for each cell type are presented as background autofluorescence controls. (**g**) The indicated cell types were infected, selected and treated as above. Cells were subjected to flow cytometric analysis and quantification of median fluorescence levels is presented.

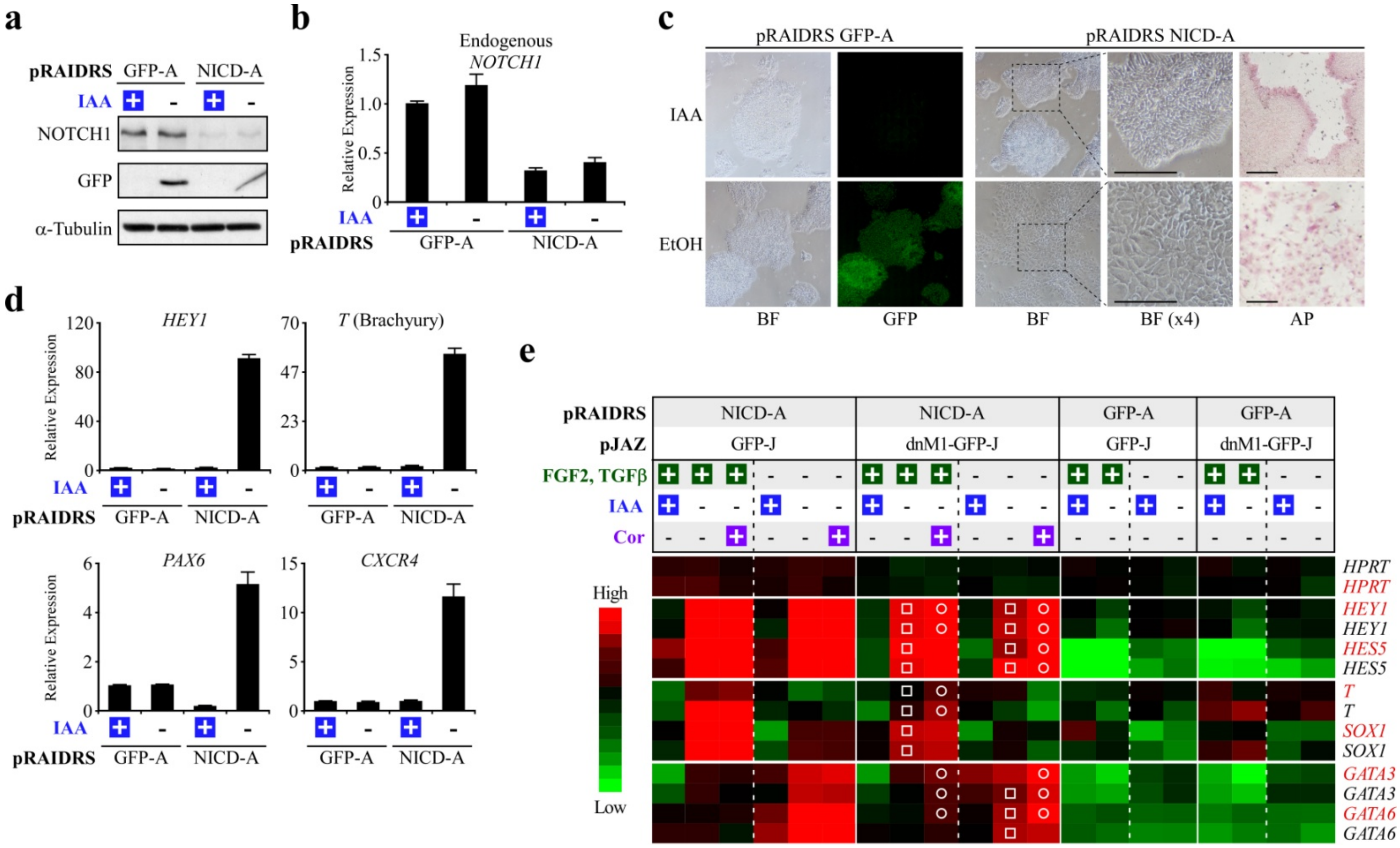

Supplementary Figure 8. A Dual Molecular Switch to Dissect NOTCH1 Function in hESCs. (**a-d**) H9 hESCs harboring pRAIDRS NLS-GFP-AID[47] (GFP-A) or pRAIDRS NICD-AID[47] (NICD-A) were maintained in mTeSR™1 media in the presence of 250 µM IAA. Cells were then washed twice and incubated for 3-5 days in the presence of 250 µM IAA (+) or 0.05% EtOH (-). (**a**) A WB analysis demonstrates the knockdown of the full-length NOTCH1 receptor (detected with the anti-NICD antibody as a protein migrating between 200 and 250 kDa) in pRAIDRS NICD cells and the accumulation of GFP-AID[47] following auxin removal. The diagonal line on the right side of the GFP blot was caused by a nick in the membrane. The accumulation of NICD-AID[47] following auxin removal is displayed in Figure 6a. α-Tubulin serves as a loading control. Un-cropped blots are displayed in Supplementary Figure 9. Experiment was performed twice and representative results are displayed. (**b**) QRT-PCR with primers that amplify only the endogenous *NOTCH1* gene demonstrates its knockdown at the mRNA level in pRAIDRS NICD-A cells. Experiment was performed 3 times and representative results are displayed. Error bars represent s.d. of 3 technical replicates. (**c**) Bright field (BF) and GFP fluorescence microscopic images, as well as microscope images of alkaline phosphatase (AP) –assayed cells, demonstrate loss of ESC morphology and AP activity in pRAIDRS NICD-A cells in the absence of auxin. 4X digitally-magnified images of the outlined areas are presented as well. Scale bars, 100 µm. (**d**) QRT-PCR analysis of selected differentiation markers, as well as of the known NOTCH1 target *HEY1*, demonstrates the induction of differentiation in pRAIDRS NICD-A cells in the absence of auxin. Experiment was performed 3 times and representative results are displayed. Error bars represent s.d of 3 technical replicates. (**e**) H9 hESCs with the indicated pRAIDRS and pJAZ constructs were cultured for 4 days with mTeSR™-E8™, which contains FGF2 and TGFβ, or mTeSR™-E6 media, which lacks FGF2 and TGFβ, and treated with 250 µM IAA and 50 µM coronatine, where indicated. QRT-PCR analysis was performed for selected genes and GAPDH-normalized values are represented as a heatmap. Two biological replicates are displayed, with the gene symbols corresponding to each repeat colored black and red. For each biological replicate, QRT-PCR analysis was performed in triplicates and heatmap represent average values. For cells harboring pRAIDRS NICD-A and pJAZ dnM1-GFP-J, white rectangles mark instances where dnMAML1-GFP-J attenuated NICD-dependent activity by at least 2 fold (compared with the expression value in pRAIDRS NICD-A pJAZ GFP-J cells under the same condition). White circles indicate instances where coronatine treatment restored NICD-A-dependent activation by at least 2 fold.

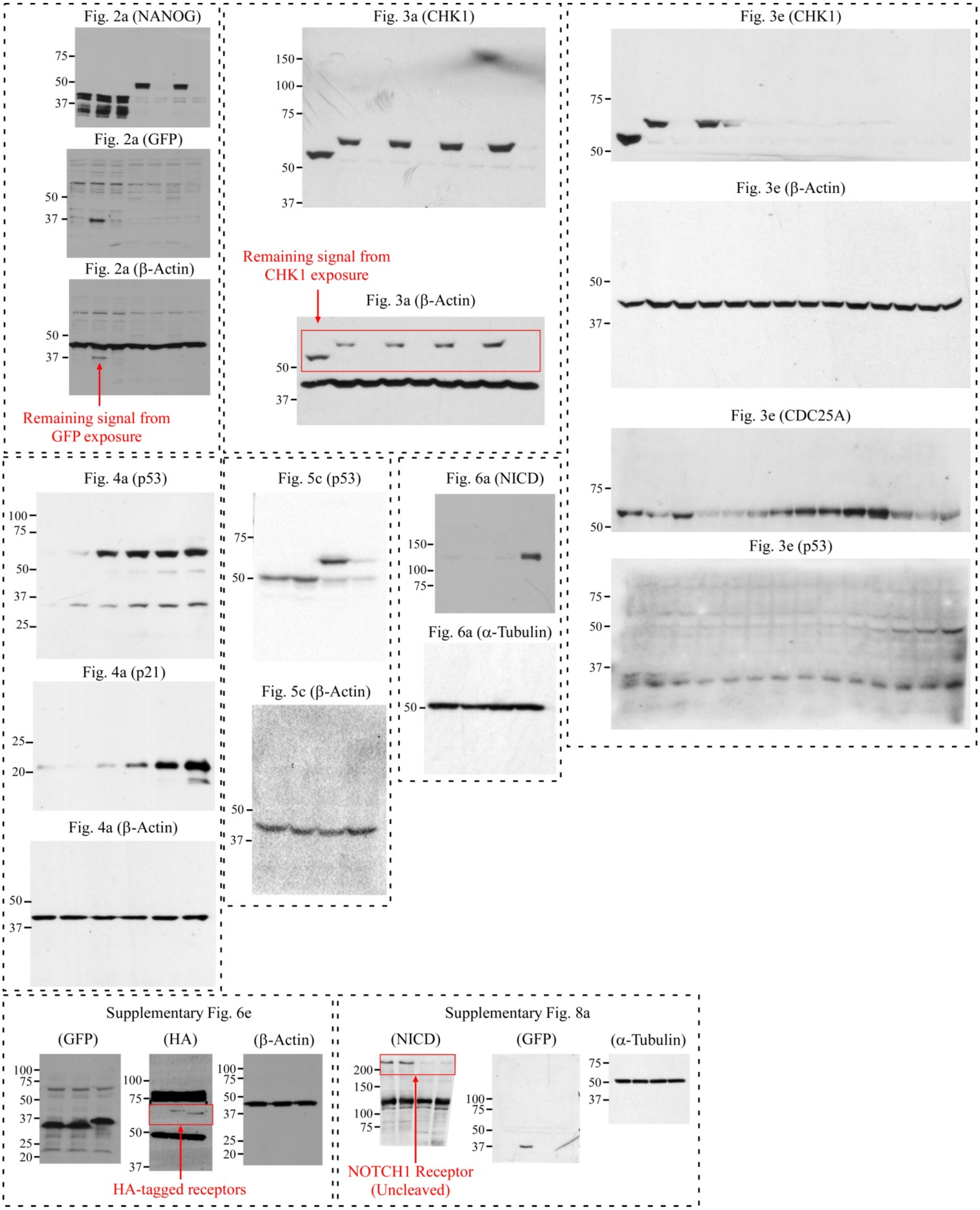

Supplementary Figure 9. Un-cropped immunoblots. Numbers on the left of each blot represent molecular weight in KDa. Note that some blots were rescanned to prepare this figure. Therefore, slight exposure differences might exist between this figure and the cropped versions displayed in the main figures.

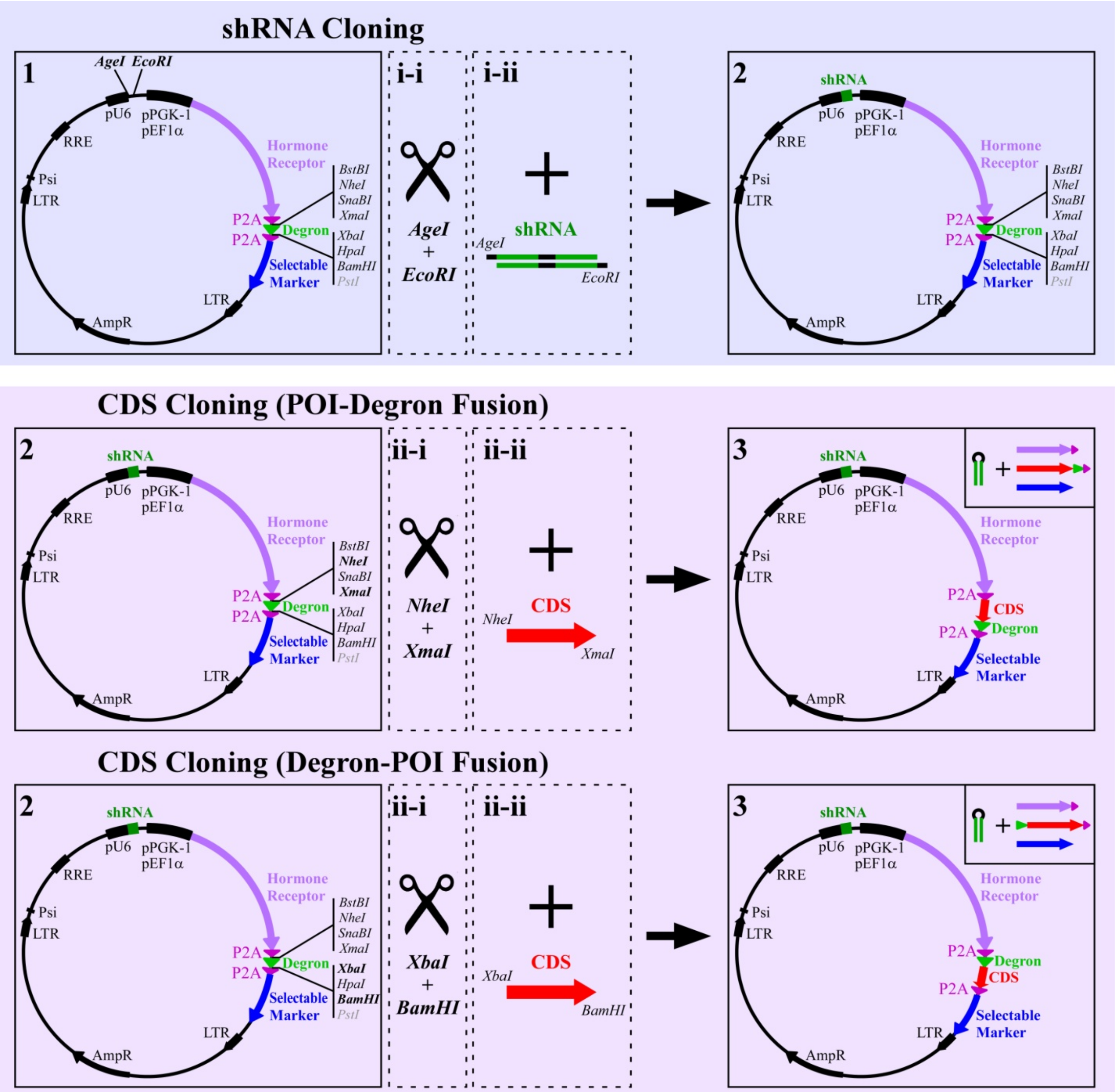

Supplementary Figure 10. The Two-Step Cloning Protocol. Strategy for constructing pRAIDRS/pJAZ rescue system plasmids. 1st step: an empty pRAIDRS/pJAZ plasmid (Box 1) is restricted with *AgeI*+*EcoRI* (step i-i), purified and ligated with a small hairpin dsDNA duplex containing *AgeI* and *EcoRI* overhangs (step i-ii), which will give rise to the shRNA. 2nd step: the POI's CDS is fused upstream or downstream of the degron, generating a POI-degron or degron-POI fusion, respectively. For example, to generate a POI-degron fusion, the shRNA-harboring plasmid (Box 2) is restricted with any desired combination of one or two REs from the 5'-MCS (*e.g.*, *NheI*+*XmaI*) (step ii-i), purified, and ligated (step ii-ii) with the POI's CDS, which was previously PCR-amplified using primers containing *NheI*+*XmaI* sites, and restricted with these *NheI*+*XmaI*. Inlet boxes depict the post-processing components harbored by each version.

<u>Notes</u>: (i) All elements expressed from the PGK-1/EF1α promoter, including the RE sites, are in-frame. Hence, the cloned CDS must not include a STOP codon or any frame-shifting elements. (ii) The size of an empty pRAIDRS/pJAZ is ~9 kb (~6 kb between LTRs). Cloning extremely long CDSs may result in oversized viral genomes, which can hinder packaging and infection. In our hands, vectors harboring CDSs of 2 kb were sufficiently infectable. (iii) We recommend testing multiple shRNA sequences before cloning the CDS. (iv) If possible, the shRNA should target the gene-of-interest's UTRs to avoid targeting of the exogenous CDS. If this is impossible, the exogenous CDS should contain 3-4 synonymous mutations in the central shRNA binding region in order to avoid targeting by the shRNA, as described by Lee *et al.*[2] (v) *PstI* is not unique in vectors containing pEF1α or *Os*COI1B^{LRR}.

Supplementary Table 1. Selected Methods to Regulate Gene Activity in Mammalian Cells.

Method	Short Description	Relevant Advantages	Relevant Disadvantages
**Chemical Inhibitors**	Small molecules that inhibit protein activity.	[1] Fast, titratable and reversible.<br>[2] Regulate protein activity.	[1] Limited mainly to enzymes.<br>[2] Low specificity.
**Genome Editing**	Various tools to alter genomic sequences. Mainly used to inactivate or modify genes. Conditional approaches are also available.	[1] Specific.<br>[2] Gene inactivation is complete.<br>[3] Flexible design.	[1] Non-titratable.<br>[2] Usually non-reversible.<br>[3] Laborious.
**RNAi**	Gene silencing by mRNA degradation or translational inhibition.	[1] Simple.<br>[2] Applicable to any gene.	[1] Low specificity (i).<br>[2] Slow (ii).<br>[3] Non-conditional.
**Conditional RNAi**	Vectors containing a conditional promoter (usually Tet-regulated) driving shRNA expression.	[1] Applicable to any gene.<br>[2] Conditional.	[1] Low specificity (i).<br>[2] Slow (ii).<br>[3] Requires rtTA/tTA (iii).
**RNAi+Tet-Ind. CDS Rescue**[3]	Lentiviral vector containing continuously-expressed shRNA and Tet-inducible CDS that rescues the phenotype exerted by the shRNA.	[1] Specific (iv).<br>[2] Conditional, reversible.<br>[3] Somewhat-titratable (v).<br>[4] Rescue system (vi).	[1] Slow response (days).<br>[2] Requires rtTA/tTA (iii).
**pAID (Auxin-Induced Degradation)**[5] (See also comment xi)	Plasmid harboring TIR1 (auxin receptor), followed by IRES and degron to which a POI is fused. The degron-fused POI is ubiquitinated and degraded following auxin treatment.	[1] Acts on protein level.<br>[2] Fast.<br>[3] Simple-to-use.<br>[4] Effective, titratable and reversible.	[1] No control over endogenous genes (vii).<br>[2] Very large degron (viii).<br>[3] Non-viral plasmid (ix).<br>[4] CMV promoter (x).<br>[5] No specialized selectable marker.<br>[6] Two plasmids for N/C -terminus fusions.
**Shield-1-Stabilized FKBP-POI**[6]	A POI is destabilized by fusion to FKBP12 variant. A small molecule (Shield-1) re-stabilizes the POI.	[1] Acts on protein level.<br>[2] Effective, titratable and reversible degradation.	[1] No control over endogenous genes (xii).<br>[2] Large degron (107-AAs).<br>[3] Relatively slow (several hours).
**pRAIDRS and pJAZ**	Each lentiviral vector is an independent rescue system containing continuously-expressed shRNA and an shRNA-immune hormone-degradable POI that rescues the phenotype exerted by the shRNA.	[1] Acts on protein level.<br>[2] Rescue system (vi).<br>[3] Specific (iv).<br>[4] Fast.<br>[5] Titratable and reversible.<br>[6] Short and stable degrons (xiii).<br>[7] Lentiviral (effective delivery).<br>[8] Independent (xiv).<br>[9] In-frame selectable marker (xv).<br>[10] Simple-to-use.<br>[11] One plasmid for N/C -terminal fusions.<br>[12] Combinatorial.	[1] Coronatine is expensive.<br>[2] Efficiency depends on protein localization (xvi).<br>[3] pJAZ ineffective in mESCs (xvii).

Comments to Supplementary Table 1:

i. RNAi can affect hundreds of genes. To overcome this, different RNAi sequences targeting the same gene can be used to substantiate causality between silencing and observed phenotypes[7].

ii. Effective gene silencing is usually obtained within 2-3 days[8, 9]. With inducible systems, silencing can be obtained within 1-2 days and reversal of the effect usually takes longer.

iii. Tet-regulated systems require rtTA or tTA, which usually necessitates the delivery of a second plasmid encoding one of these proteins, or a specialized cell type that stably expresses it.

iv. If an RNAi-dependent phenotype is reversed by the CDS of the silenced gene, it is unlikely that the phenotype stemmed from an RNAi off-target effect[7].

v. Tet-induced expression is usually hard to accurately titrate.

vi. A rescue (or genetic complementation) system enables the replacement of an endogenous gene with an exogenous version that can be regulated externally. Represents a type of a molecular switch.

vii. pAID allows expression of auxin-degradable proteins. However, it does not contain a component, such as an shRNA, that inactivates an endogenous gene and allows its replacement by the auxin-degradable protein.

viii. The AID degron used in pAID is the full-length *A. thaliana* IAA17 transcription factor. As a degron, it suffers from several disadvantages, including its large size (228-AAs), its ability to confer nuclear localization to the fused POI (data

not shown) and, possibly, a tendency to be spontaneously cleaved-off from the POI. Notably, the observation of spontaneous cleavage of GFP-AID[228] in pRAIDRS-infected 293T cells (Supplementary Figure 1) was not reported by other groups, who successfully used GFP-AID[228].

ix. Non-viral plasmids are hard to deliver into some types of mammalian cells, including hard-to-transfect cells (such as fibroblasts, ESCs and many primary cell types) and slow-proliferating cells. Additionally, genomic integration of non-viral plasmids is an extremely rare event in many cell types.

x. The CMV promoter undergoes silencing in certain mammalian stem cells, such as human ESCs[10, 11].

xi. Auxin-dependent degradation was utilized successfully in several studies of mammalian cells[12, 13, 14, 15], albeit not in stem cells. Nevertheless, to engineer auxin-degradable proteins in mammalian cells the authors had to use several consecutive and rather-laborious steps. For example, Holland *et al.*[13] as well as Han *et al.*[12] first generated cell lines that overexpress the TIR1 receptor, then overexpressed an AID-fused POI, and finally transiently knocked-down the gene encoding for the endogenous POI. Similarly, Lambrus *et al.*[14] sequentially targeted the AID degron to the two endogenous alleles of the POI, and then overexpressed TIR1.

xii. The system is limited to exogenously-expressed proteins, unless genome-editing is used to fuse the destabilizing FKBP domain to both alleles of an endogenous gene, or a form of rescue system is established by inactivating the endogenous gene and replacing it with an FKBP-fused version.

xiii. pRAIDRS and pJAZ harbor relatively short degrons (47-AAs and 33-AAs, respectively). Shorter degrons reduce the likelihood of steric interference with the POI's function, and supposedly, have less non-degron functions (such as interactions with additional proteins or DNA or effect on protein localization). Additionally, the shorter AID degron seem to suffer less from spontaneous cleavage from the POI compare with the full-length AID[228], at least in our experimental settings (Supplementary Fig. 1c,d).

xiv. As opposed to the RNAi+Tet-Inducible CDS Rescue System, pRAIDRS and pJAZ contain all the necessary genetic elements to silence an endogenous gene and replace it by a hormone-degradable POI. They can be used in non-specialized cells without additional components.

xv. In pRAIDRS and pJAZ the selectable markers (either PuroR or BSD genes) are cloned in-frame with the hormone receptor and degron-POI. Following translation and cleavage at the P2A peptides, the selectable marker is released and can render cells resistant to its corresponding drug. The expression of all components from a single promoter and as a single pre-processed protein reduces the likelihood that in drug-resistant cells will silencing or mutation the hormone receptor or degron-POI will occur.

xvi. Both pRAIDRS and pJAZ show increased efficiency with nuclear POIs compared to cytoplasmic POIs.

xvii. Coronatine-dependent degradation in mouse embryonic stem cells is ineffective (usually 50-80%, compared with >90% in other mouse cell types and all tested human cell types).

Supplementary Table 2. Cloning Primers.

#	Name	Primer Sequence (5' to 3')
1	F-Box-RF-F1	cagggggatcgtcgacgccaccatgacctacttccccgagg
2	F-Box-RF-R1	gggccattgtcacatgctcgcggctcagtctctcgatctcg
3	JAZ1-31-F1-*XmaI*	atattacccgggcctacacctctgacagagctgcctatcgccag
4	JAZ1-31-R1-*XbaI*	atactatctagaaggagccttgctggtcactctgtccttccgc
5	JAZ1-FL-F1-*XmaI*	acgtggcccgggatgtcgagttctatggaatg
6	JAZ1-FL-R1-*XbaI*	cgcggctctagatatttcagctgctaaaccgag
7	*Os*JAZ-33-F1	tatattcccgggcacgccgctgccctgcctgagatgcctatcgccag
8	*Os*JAZ-33-R1	tgactgtctagatggctcgcttgtggtggtgattctgtgcttccgc
9	HA-*Os*Tir1-F1	gtctgagtcgacgccaccatgtacccatacgatgttccagattacgctacctacttccccgaggaag
10	P2A-*BstBI*-R	atcttattcgaaggggccggggttctc
11	F-Box-RF-F3	cagggggatcgtcgacgccaccatgtacccatacgatgttccag
12	F-Box-RF-R3	aaggcacggtcacgtgctttctgctcagtctctcgatctcg
13	*NheI*-NLS-GFP-F	gcagccgctagcccaaaaaagaaaagaaaagttatggtgagcaagggcgaggag
14	GFP-R2-*XmaI*	gatgtgcccgggcttgtacagctcgtccatgcc
15	mOrange-R1-*XmaI*	atcagtcccgggcttgtacagctcgtccatgc
16	GFP-F2-*NheI*	gatgtggctagcatggtgagcaagggcgaggag
17	mOrange-*NheI*-F1	atcagagctagcatggtgagcaagggcgaggag
18	F-Box-RF-F2	ggggatcgtcgacgccaccatggtttcatgggactcccttcc
19	F-Box-RF-R2	ttgtcacatgctcgcgtgtctcagacgctaggcgatacca
20	*Hs*Skp1-RF-F	cagggggatcgtcgacgccaccatgccttcaattaagttgcagagt
21	*Hs*Skp1-RF-R(a)	ccacttcctcggggaagtaggtcttctcttcacaccactggt
22	*Hs*Skp1-RF-R(b)	gggccattgtcacatgctcgcgcttctcttcacaccactggt
23	*Hs*Skp1-RF-R(c)	ccacttcctcggggaagtaggtccccttgatcatattggcaaca
24	*Hs*Skp1-RF-R(d)	gggccattgtcacatgctcgcgccccttgatcatattggcaaca
25	pEF1α-RF-F	actttggccgcggctcgaggggctccggtgcccgtcag
26	pEF1α-RF-R	catggtggcgtcgacgatcccctcacgacacctgaaatggaa
27	mNanog-F1-*XbaI*	tgtcagtctagaatgagtgtgggtcttcctgg
28	mNanog-R1-*BamHI*	tgtcagggatcctatttcacctggtggagtc
29	mp53-F2-*BstBI*	tgtcagttcgaaatgactgccatggaggagtc
30	mp53-R2-*NheI*	tgtcaggctagcgtctgagtcaggccccactt
31	mChk1-F1-*XbaI*	ggtcagtctagaatggcagtgccttttgtgg
32	mChk1-R1-*BamHI*	ggtcagggatcctgtaacaggaaaccaaacc
33	mut*AgeI*-F3	gaggggtcggcaattgaagcggtgcctagagaaggtg
34	mut*AgeI*-R3	caccttctctaggcaccgcttcaattgccgacccctc
35	FLAG-Tir1-F1	gtctgagtcgacgccaccatggactacaaagacgatgacgacaagacctacttccccgaggaag
36	3Myc-Tir1-F1	gtctgagtcgacgccaccatggagcagaaactcattagcgaggaggacctgaacagcgaacagaaactcatttccgaagaggatctcaa ctccgagcagaagctgatcagcgaggaggacctgagatccacctacttccccgaggaag
37	hNICD-F1-*NheI*	attctagctagcatgcggcggcagcatggccag
38	hNICD-R3-*SnaBI*	ctcggatacgtacttgaaggcctccggaatgc
39	hdnMAML1-F2-*BstBI*	attctattcgaactgccgcggcacagcgcggtc
40	hdnMAML1-R2-NheI	attaaagctagcgtgcttcccggcgcgcttgg
41	hp53-F1-*XbaI*	agcttctagaatggaggagccgcagtcag
42	hp53-R1-*BamHI*	agctggatccgtctgagtcaggcccttctg

Supplementary Table 3. shRNA Oligonucleotides.

#	Name	Primer Sequence (5' to 3')
101	mNanog-shRNA-F	ccgggccaacctgtactatgtttaactcgagttaaacatagtacaggttggctttttg
102	mNanog-shRNA-R	aattcaaaaagccaacctgtactatgtttaactcgagttaaacatagtacaggttggc
103	mChk1-shRNA-F	ccggcccatgtagtagtatcactttctcgagaaagtgatactactacatgggttttt
104	mChk1-shRNA-R	aattaaaaacccatgtagtagtatcactttctcgagaaagtgatactactacatggg
105	hNOTCH1-shRNA-F	ccggctttgtttcaggttcagtattctcgagaatactgaacctgaaacaaagtttttg
106	hNOTCH1-shRNA-R	aattcaaaaactttgtttcaggttcagtattctcgagaatactgaacctgaaacaaag
107	Luciferase-shRNA-F	ccggcttacgctgagtacttcgactcgagtcgaagtactcagcgtaagtttttg
108	Luciferase-shRNA-R	aattcaaaaacttacgctgagtacttcgactcgagtcgaagtactcagcgtaag
109	hp53-shRNA-F	ccgggagggatgtttgggagatgtactcgagtacatctcccaaacatccctctttttg
110	hp53-shRNA-R	aattcaaaaagagggatgtttgggagatgtactcgagtacatctcccaaacatccctc

Supplementary Table 4. QRT-PCR Primers.

**Target**	**Forward Primer Sequence (5'-3')**	**Reverse Primer Sequence (5'-3')**
Human *GAPDH*	acccactcctccacctttga	ctgttgctgtagccaaattcgt
Human *HPRT1*	gaccagtcaacaggggacat	cctgaccaaggaaagcaaag
Human *MDM2*	gaatcatcggactcaggtacatc	tctgtctcactaattgctctcct
Human p21 (*CDKN1A*)	tgtccgtcagaacccatgc	aaagtcgaagttccatcgctc
Human *HEY1*	aggagagtgcggacgagaat	aacctagagccgaactcaagt
Human *HES5*	accgcatcaacagcagcat	gaaggctttgctgtgcttcag
Human *T* (Brachyury)	cagtggcagtctcaggttaagaagga	cgctactgcaggtgtgagcaa
Human *SOX1*	tttcccctcgctttctca	tgcaggctgaattcggtt
Human *GATA3*	gcccctcattaagcccaag	ttgtggtggtctgacagttcg
Human *GATA6*	gcgggctctacagcaagatg	acagttggcacaggacaatcc
Human *NANOG*	cctgaagacgtgtgaagatgag	gctgattaggctccaaccatac
Human *PAX6*	aggtattacgagactggctcc	tcccgcttatactgggctattt
Human *CXCR4*	atgaaggaaccctgtttccgt	agatgatggagtagatggtggg
Human Endogenous *NOTCH1*	cctgcccgttcttgaaatgt	ggagcatcttcttcggaacct
Mouse *Cdx2*	caaggacgtgagcatgtatcc	gtaaccaccgtagtccgggta
Mouse *Cxcl12*	tgcatcagtgacggtaaacca	ttcttcagccgtgcaacaatc
Mouse *Esrrb*	caggcaaggatgacagacg	gagacagcacgaaggactgc
Mouse *Fas* (CD95, APO-1)	tatcaaggaggcccattttgc	tgtttccacttctaaaccatgct
Mouse *Gapdh*	agaacatcatccctgcatcc	cacattgggggtaggaacac
Mouse *Gata4*	ccctacccagcctacatgg	acatatcgagattggggtgtct
Mouse *Gata6*	ttgctccggtaacagcagtg	gtggtcgcttgtgtagaagga
Mouse *Hand1*	cccctcttccgtcctcttac	ctgcgagtggtcacactgat
Mouse *Mdm2*	tgtctgtgtctaccgagggtg	tccaacggactttaacaacttca
Mouse *Mixl1*	atccgcccggaccctccaaa	tcggttctggaaccacacctgga
Mouse Nestin (*Nes*)	aggagaagaagaaccaagaatggagga	tcggcttctggacctcccagt
Mouse Noxa (*Pmaip1*)	aaaagagcaggatgaggagcc	gtccttcaagtctgctggcac
Mouse p21 (*Cdkn1a*)	cctggtgatgtccgacctg	ccatgagcgcatcgcaatc
Mouse *Pax3*	gcagcgcaggagcagaacca	gcactcgggcctcggtaagc
Mouse *Sox1*	atgcaccgctacgacatggg	gctccgacttgaccagagatcc
Mouse *Sox17*	cgcacggaattcgaacagta	gtcaaatgtcggggtagttg
Mouse *Sox7*	cccccgaccttcaggggacaag	ggacagtgtcagcgccttccat
Mouse *T* (Brachyury)	gcttcaaggagctaactaacgag	ccagcaagaaagagtacatggc
Mouse *Tcl1*	aaattccaggtgatcttgcg	tgtccttggggtacagttgc
eGFP-1	agccgctaccccgaccacat	cggttcaccagggtgtcgcc
eGFP-2	gacggcgacgtaaacggcca	cagcttgccggtggtgcaga

Supplementary References

1. Calderón Villalobos LIA*, et al.* A combinatorial TIR1/AFB–Aux/IAA co-receptor system for differential sensing of auxin. *Nat Chem Biol* **8**, 477-485 (2012).

2. Lee DF, Su J, Sevilla A, Gingold J, Schaniel C, Lemischka IR. Combining competition assays with genetic complementation strategies to dissect mouse embryonic stem cell self-renewal and pluripotency. *Nat Protoc* **7**, 729-748 (2012).

3. Ivanova N*, et al.* Dissecting self-renewal in stem cells with RNA interference. *Nature* **442**, 533-538 (2006).

4. Lee HY*, et al.* Oryza sativa COI homologues restore jasmonate signal transduction in Arabidopsis coi1-1 mutants. *PLoS ONE* **8**, e52802 (2013).

5. Nishimura K, Fukagawa T, Takisawa H, Kakimoto T, Kanemaki M. An auxin-based degron system for the rapid depletion of proteins in nonplant cells. *Nat Methods* **6**, 917-922 (2009).

6. Banaszynski LA, Chen LC, Maynard-Smith LA, Ooi AG, Wandless TJ. A rapid, reversible, and tunable method to regulate protein function in living cells using synthetic small molecules. *Cell* **126**, 995-1004 (2006).

7. Cullen BR. Enhancing and confirming the specificity of RNAi experiments. *Nat Methods* **3**, 677-681 (2006).

8. Brummelkamp TR, Bernards R, Agami R. A system for stable expression of short interfering RNAs in mammalian cells. *Science* **296**, 550-553 (2002).

9. van de Wetering M*, et al.* Specific inhibition of gene expression using a stably integrated, inducible small-interfering-RNA vector. *EMBO reports* **4**, 609-615 (2003).

10. Xia X, Zhang Y, Zieth CR, Zhang SC. Transgenes delivered by lentiviral vector are suppressed in human embryonic stem cells in a promoter-dependent manner. *Stem cells and development* **16**, 167-176 (2007).

11. Norrman K, Fischer Y, Bonnamy B, Wolfhagen Sand F, Ravassard P, Semb H. Quantitative comparison of constitutive promoters in human ES cells. *PLoS ONE* **5**, e12413 (2010).

12. Han JS, Holland AJ, Fachinetti D, Kulukian A, Cetin B, Cleveland DW. Catalytic assembly of the mitotic checkpoint inhibitor BubR1-Cdc20 by a Mad2-induced functional switch in Cdc20. *Mol Cell* **51**, 92-104 (2013).

13. Holland AJ, Fachinetti D, Han JS, Cleveland DW. Inducible, reversible system for the rapid and complete degradation of proteins in mammalian cells. *Proc Natl Acad Sci U S A* **109**, E3350-3357 (2012).

14. Lambrus BG*, et al.* p53 protects against genome instability following centriole duplication failure. *The Journal of cell biology* **210**, 63-77 (2015).

15. Rodriguez-Bravo V*, et al.* Nuclear Pores Protect Genome Integrity by Assembling a Premitotic and Mad1-Dependent Anaphase Inhibitor. *Cell* **156**, 1017-1031 (2014).
